# Targeting carbonic anhydrase IX/XII prevents the anti‐ferroptotic effect of stromal lactic acid in prostate carcinoma

**DOI:** 10.1002/1878-0261.70083

**Published:** 2025-06-26

**Authors:** Elisa Pardella, Giuseppina Comito, Luigi Ippolito, Erica Pranzini, Marta Iozzo, Giulia Gangarossa, Francesca Virgilio, Silvia Bua, Alessio Nocentini, Giada Sandrini, Nicla Lorito, Marina Bacci, Gabriella Nesi, Pietro Spatafora, Sergio Serni, Claudiu T. Supuran, Andrea Morandi, Paola Chiarugi, Elisa Giannoni

**Affiliations:** ^1^ Department of Experimental and Clinical Biomedical Sciences “Mario Serio” University of Florence Italy; ^2^ Department of NEUROFARBA, Pharmaceutical and Nutraceutical Section University of Florence Italy; ^3^ Institute of Oncology Research (IOR) Università della Svizzera Italiana (USI) Bellinzona Switzerland; ^4^ Section of Pathological Anatomy, Department of Health Sciences University of Florence Italy; ^5^ Unit of Urological Robotic Surgery and Renal Transplantation, Careggi Hospital University of Florence Italy; ^6^ Department of Experimental and Clinical Medicine University of Florence Italy

**Keywords:** cancer‐associated fibroblasts, carbonic anhydrase, ferroptosis, lactic acid, prostate cancer, tumor microenvironment

## Abstract

Ferroptosis is a form of regulated cell death dependent on iron‐driven phospholipid peroxidation and is controlled by both cell autonomous and non‐cell autonomous mechanisms. In prostate cancer (PCa), tumor cells engage in a metabolic crosstalk with cancer‐associated fibroblasts (CAFs), resulting in increased utilization of CAF‐secreted lactic acid, that ultimately supports cancer aggressiveness. In this context, the effect of the prostate tumor microenvironment in modulating ferroptosis sensitivity has not yet been extensively investigated. Here, we demonstrate that CAF‐secreted lactic acid protects PCa cells from ferroptosis induction and supports the upregulation of the antioxidant enzyme glutathione peroxidase 4 (GPX4). Interestingly, targeting carbonic anhydrase IX/XII (CA IX/XII), the main regulators of microenvironmental acidosis, in tumor and stromal compartments hinders lactic acid shuttle within the tumor–stroma interplay and thus, prevents ferroptosis resistance induced by lactic acid. Analyses of tissue samples from PCa patients also revealed that GPX4, CA IX, and CA XII expression levels increase during PCa progression. Overall, these findings support a role for stromal lactic acid in mediating ferroptosis resistance in PCa, identifying CA IX/XII as potential therapeutic targets regulating ferroptosis sensitivity.

## Graphical abstract

In prostate carcinoma, lactic acid, secreted by highly glycolytic cancer‐associated fibroblasts, is imported into tumor cells through the MCT1 transporter and prevents RSL3 and erastin‐induced ferroptosis (A). Targeting of carbonic anhydrase IX/XII, the main extracellular pH regulators, in tumor and stromal cells reduces microenvironmental acidosis and impairs lactic acid uptake by cancer cells, thereby sensitizing them to ferroptosis (B).

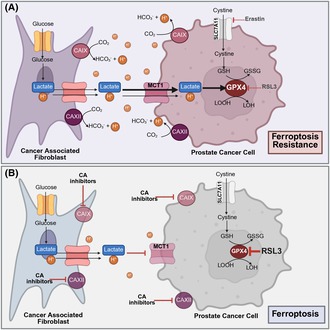

**Abbreviations**
ANOVA: analysis of variance
ATCC: American Type Culture Collection
BCA: bicinchoninic acid
CA: carbonic anhydrase
CAFs: cancer associated fibroblasts
CA IX: carbonic anhydrase isoform IX
CA XII: carbonic anhydrase isoform XII
CM: conditioned media
CRPC: castration‐resistant prostate cancer
DAB: 3′,3‐diaminobenzidine
DFOM: deferoxamine mesylate salt
DMEM: Dulbecco's Modified Eagle's Medium
FBS: fetal bovine serum
FIN: ferroptosis inducer
GPX4: glutathione peroxidase 4
GSH: reduced glutathione
GSSG: oxidized glutathione
HIF1α: hypoxia‐inducible factor 1‐alpha
HPFs: healthy prostate fibroblasts
IHC: immunohistochemistry
MCT1i: MCT1 inhibitor
MCTs: monocarboxylate transporters
MFI: mean fluorescence intensity
MUFA: monounsaturated fatty acid
NFS1: cysteine desulfurase
PCa: prostate cancer
PDAC: pancreatic ductal adenocarcinoma
PUFA: polyunsaturated fatty acid
ROS: reactive oxygen species
SEM: standard error of the mean
SLC1A5: solute carrier family 1 member 5
SLC7A11: solute carrier family 7 member 11
TMA: tissue microarray
TME: tumor microenvironment

## 1 Introduction

Metabolic interactions between cellular components of the tumor microenvironment (TME) are finely tuned to support tumor cell biosynthetic and bioenergetic requirements, dampen immune responses, and confer anticancer therapy resistance [1, 2]. Of note, in several tumors, including prostate cancer (PCa), intercellular contacts between stromal and tumor cells increase glycolytic flux in cancer‐associated fibroblasts (CAFs), resulting in enhanced lactic acid secretion [3, 4, 5]. Lactic acid represents one of the most abundant metabolites across diverse cancer types [6, 7, 8], correlating with tumor aggressiveness, and it is emerging as a central hub for bioenergetics, epigenetics, and immunosurveillance [9, 10, 11, 12, 13, 14]. PCa cells exploit CAF‐secreted lactic acid to sustain an extensive metabolic reprogramming, functional to the acquisition of an invasive phenotype [4, 15, 16, 17]. Mechanistically, stromal lactic acid fosters tricarboxylic acid cycle metabolism by increasing mitochondrial activity [15] and contributes to lipid biosynthesis, supporting histone acetylation [16]. Lactic acid production by glycolytic cells also leads to the generation of an acidic TME. Indeed, the enhanced metabolic demands of Warburg‐dependent cells, in the presence of disorganized tumor vasculature, result in increased production of metabolic acids (e.g., lactate and H^+^) [18]. To avoid intracellular acidification, lactic acid is transported across cell membranes by monocarboxylate transporters (MCTs, i.e., MCT1‐4), acting as symporters of dissociated lactate^−^ and H^+^ [19]. Extracellular acidification strongly promotes tumor growth and metastasis, suppresses immunity and sustains tumor metabolic reprogramming [20, 21]. Among the different transporters and enzymes involved in the maintenance of pH homeostasis in tumor cells [22], carbonic anhydrases (CAs), a family of zinc‐metalloenzymes catalyzing the reversible hydration of CO_2_ to ${\mathrm{HCO}}_{3}^{-}$ and H^+^, plays a pivotal role [23, 24, 25]. In particular, the transmembrane isoforms CA IX and CA XII act as crucial pH regulators of the TME, as their catalytic sites are located in the extracellular domain [26, 27, 28], and are considered anticancer targets [22, 29]. Besides being mainly expressed by tumor cells, the stabilization of hypoxia‐inducible factor 1‐alpha (HIF1α) also causes CA IX overexpression in prostate CAFs, leading to the epithelial–mesenchymal transition in tumor cells [30, 31]. Tumor acidity as well as specific TME cellular components and the abundance/deprivation of selected nutrients can either promote or decrease the susceptibility to ferroptosis in cancer cells [32, 33]. Ferroptosis is a form of regulated and nonapoptotic cell death, driven by iron‐dependent extensive peroxidation of polyunsaturated fatty acid (PUFA)‐containing phospholipids at cell membranes [34, 35, 36]. Ferroptosis is characterized by three main hallmarks: (a) deregulated antioxidant machinery; (b) availability of iron as labile pool for initiating the Fenton reaction; (c) altered ratio between monounsaturated fatty acids (MUFAs) and highly oxidizable PUFAs in membrane phospholipids [37, 38, 39]. In PCa, ferroptosis inducers (FINs) have recently emerged as potential novel therapeutic approaches for treating the advanced stages of the disease [40, 41]. However, the effects of the prostate TME on FIN efficacy have not been elucidated yet. Here, we prove that CAF‐secreted lactic acid acts as a negative regulator of ferroptosis sensitivity in PCa and interfering with extracellular‐facing CA IX/XII in both stromal and tumor compartments prevents ferroptosis resistance in PCa cells exposed to stromal lactic acid. Moreover, we underline that glutathione peroxidase 4 (GPX4), one of the main negative ferroptosis regulators converting lipid peroxides into nontoxic alcohols, is upregulated following lactic acid exposure, and in PCa tissues its protein levels positively correlate with MCT1 expression and increase during PCa progression.

## 2 Materials and methods

### 2.1 Cell lines and culture

Human metastatic and castration‐resistant prostate cancer (CRPC) cell lines PC3 (RRID: CVCL_0035) and DU145 (RRID: CVCL_0105) were obtained from American Type Culture Collection (ATCC). Human healthy prostate fibroblasts (HPFs) were isolated from surgical explants of patients affected by benign prostate hyperplasia, while CAFs were isolated from surgical explants of patients subjected to surgical intervention for prostate cancer. The study and the methodologies were approved by the local Ethics Committee (Comitato Etico Regionale per la Sperimentazione Clinica della Regione Toscana, Area Vasta Centro, CEAVC‐2018‐256). The samples were collected from November 2019 to November 2022 at the Azienda Ospedaliera Universitaria (AOU) Careggi (Florence, Italy) and processed at the Department of Experimental and Clinical Biomedical Sciences “Mario Serio,” University of Florence (Florence, Italy). The experiments were undertaken with the understanding and written consent of each subject. The study methodologies were conformed to the standards set by the Declaration of Helsinki. Prostate cancer cell lines and isolated primary cells were cultured in phenol‐red Dulbecco's Modified Eagle's Medium (DMEM, Euroclone, Pero, Milan, Italy, #ECB7501L) supplemented with 10% Fetal Bovine Serum (FBS, Euroclone #ECS5000L), 2 mm l‐Glutamine (Merck Sigma, Darmstadt, Germany, #G7513‐100ML) and 1% penicillin/streptomycin (Merck Sigma #P0781‐100 ML) at 37 °C and 5% CO_2_ in a humidified atmosphere. The PC3 and DU145 cell lines were obtained from ATCC within the last 3 years and authenticated by STR profiling by the supplier. Cells were routinely and rigorously monitored for the maintenance of their characteristics, such as morphology, cell doubling time and cell growth features. All cell lines were routinely tested for Mycoplasma contamination using the MycoAlert Mycoplasma Detection kit (Lonza, Basel, Switzerland, #LOLT07710).

### 2.2 Conditioned media isolation from primary cells

HPFs and CAFs were incubated for 48 h with serum‐free medium. The resulting conditioned media (HPF‐CM or CAF‐CM) were then collected, centrifuged, filtered with 0.22‐μm filters, and used freshly or stored at −80 °C. When specified, CM were boiled at 95 °C for 20 min to eliminate protein components.

### 2.3 Cell treatments and reagents

PC3 and DU145 cells were incubated for 48 h with serum‐free medium, or with CM isolated from HPFs or CAFs, or with serum‐free medium supplemented with 20 mm lactic acid (Sigma‐Aldrich, St. Louis, MO, USA, #L6402). For the treatment with sodium lactate or acidic conditions, DU145 cells were incubated for 48 h with 20 mm sodium l‐lactate (Sigma‐Aldrich, #L7022) or acidic medium (pH 6.5), that was obtained as previously described with modifications [42]. Briefly, HCl 1 N was progressively added to the serum‐free medium until the pH value stabilized at pH 6.5 ± 0.1. pH was monitored with the micropH combination electrode (Sigma‐Aldrich #Z113425). The following compounds were used: RSL3 ((1S, 3R)‐RSL3, #S1855), Erastin (#S7242), Ferrostatin‐1 (#S7243) and Liproxstatin‐1 (#S7699) were purchased from Selleckchem (Houston, TX, USA); deferoxamine mesylate salt (DFOM, #D9533) was purchased from Sigma‐Aldrich; MCT1 inhibitor (MCT1i) AR‐C155858 (#HY‐13248) was purchased from MedChemExpress, Monmouth Junction, NJ, USA. Carbonic anhydrase inhibitors SLC‐0111, CAI#1, and CAI#2 were previously developed and described [43, 44, 45, 46]. The compound concentrations and the timing of the treatments are specified in the Figure Legends for each experiment.

### 2.4 RNA sequencing analysis

To investigate the effect of lactic acid in DU145 cell line, RNA‐sequencing data (available with GSE195639 number) provided by Ippolito et al. [16] were analyzed. Three biological replicates of DU145 cells exposed to lactic acid were compared to four biological replicates of untreated DU145 cells according to the DESeq pipeline in R environment, revealing 2151 differentially regulated genes (|log2FC| > 0.5 and adjusted *P* value < 0.05). To assess the involvement of lactic acid‐regulated genes in the ferroptosis process, the FerrDb database [47] was exploited. Ferroptosis regulators listed in FerrDb database, including 255 drivers and 208 suppressors, were intersected with significantly differentially regulated genes under lactic acid exposure, according to RNA‐seq results. The ferroptosis driver genes that were downregulated under lactic acid conditions and the ferroptosis suppressor genes that were upregulated under lactic acid conditions were considered for downstream analysis. Subsequently, VST‐normalized expression values of the 19 analyzed genes were visualized using a heatmap, distinguishing between the significantly upregulated and downregulated genes under lactic acid conditioning.

### 2.5 Patient database

A comprehensive database of PCa patients that includes 174 normal samples and 316 CRPC cases was used [48]. VST‐normalized expression data and its annotations were downloaded from the Zenodo repository [49]. To compare GPX4 expression levels in CRPC and NORMAL samples, the Wald test from the deseq package was performed on VST‐normalized expression values. Disease‐free survival was evaluated in the TCGA‐PRAD Cohort that includes 492 patients, and the graph was created using the gepia software tool [50].

### 2.6 Protein extraction and western blot

PC3 and DU145 cells were incubated with serum‐free medium (CTR), 20 mm lactic acid, or CAF‐CM for 48 h. Then, cells were lysed in ice‐cold RIPA lysis buffer (Thermo Fisher Scientific, Waltham, MA, USA, #8990), supplemented with protease inhibitors (Sigma‐Aldrich, #P8340) and phosphatase inhibitors (Sigma‐Aldrich, #P0044). Cell lysates were clarified by centrifugation at 14 000 ***g*** for 10 min at 4 °C. Proteins were quantified by bicinchoninic acid (BCA) assay (Sigma‐Aldrich, #1003579336). 10–25 μg of total proteins were loaded on 4–20% acrylamide precast SDS/PAGE gels (BioRad, Hercules, CA, USA, #4568093 – #4568096) and then transferred on polyvinylidene difluoride (PVDF) membranes using the Trans‐Blot Turbo Transfer Pack (BioRad, #1704157). After incubation for 1 h at room temperature with blocking buffer (PBS with 0.1% Tween 20 (Sigma‐Aldrich, #P1379) (PBS‐T) containing 5% nonfat dry milk), immunoblots were incubated overnight at 4 °C with primary antibodies diluted 1 : 1000 in PBS‐T with 5% nonfat dry milk. The primary antibodies used were: GPX4 (Cell Signaling, Leiden, The Netherlands, rabbit, #BK52455) and HSP90 (Santa Cruz Biotechnology, Dallas, TX, USA, mouse, #sc‐69703). The following day, membranes were incubated for 1 h at room temperature with appropriate horseradish peroxidase‐conjugated secondary antibodies diluted 1 : 5000 in PBS‐T containing 5% non‐fat dry milk. The secondary antibodies used were: anti‐rabbit (Santa Cruz Biotechnology, #sc‐2357) and anti‐mouse (Santa Cruz Biotechnology, #sc‐516102). Antibodies were detected using the Clarity Western ECL Substrate (BioRad, #1705061) or the Clarity Max ECL substrate (BioRad, #1705062) at the Amersham Imager 600 luminometer (Amersham, Buckinghamshire, UK). Densitometric analysis of western blot images was performed with imagej software, National Institute of Health, Bethesda, MD, USA, https://imagej.net/ij/.

### 2.7 Immunohistochemistry (IHC)

To analyze protein expression in PCa tissues, a tissue microarray (TMA, US Biomax, Derwood, MD, USA, #PR633a) was used. The TMA was stained with the following antibodies: GPX4 (Invitrogen‐Thermo Fisher Scientific, Waltham, MA, USA, rabbit, #PA5‐102521, 1 : 300), Carbonic Anhydrase IX (CA IX) EP161 (Cell Marque, Rocklin, CA, USA, rabbit, #379R‐14, 1 : 100), Carbonic anhydrase XII (CA XII) (AbCam, rabbit, #ab195233, 1 : 100), and MCT1 (AbCam, Cambridge, UK, mouse, #ab85021, 1 : 300). The specificity of the antibodies was tested using negative and positive tissue samples. The Leica BOND‐RXm automated system (Leica Microsystem) was used for IHC analyses. Slides were developed with 3',3‐diaminobenzidine (DAB) or Fast Red (Leica Microsystem, Wetzlar, Germany) and counterstained with hematoxylin. The antibody staining intensity for each core of the TMA was associated with an intensity histoscore (H‐score) of 0, +1, +2, +3, evaluated by at least two researchers. Images were obtained with a slide scanner (Aperio LV1, Leica Biosystems) and analyzed with the imagescope Software (Leica Microsystem).

### 2.8 GSH/GSSG measurements

DU145 and PC3 cells were seeded in 24‐well plates (2–3 × 10^4^ cells per well) and treated for 48 h with serum‐free medium (control) or 20 mm lactic acid. Total glutathione (GSH + GSSG) and oxidized glutathione (GSSG) content were measured after conversion to reduced glutathione (GSH), whose amount is directly proportional to the luminescence signal (in relative light units, RLU), quantified with a luminometer (Biotek Agilent, Santa Clara, CA, USA, Sinergy H1). Then, the GSH/GSSG ratio was calculated from luminescence measurements, according to the manufacturer's instructions (Promega, Madison, WI, USA, GSH/GSSG‐Glo Assay #V6611).

### 2.9 NADPH/NADP ^+^ measurements

DU145 cells were seeded in 24‐well plates (2 × 10^4^ cells per well) and treated for 48 h with serum‐free medium (control) or 20 mm lactic acid. Then, the NADPH/NADP^+^ ratio was obtained according to manufacturer's instructions (Promega, NADPH/NADP‐Glo Assay #G9081) and calculated from the luminescence signal (in relative light units, RLU), measured using a luminometer (Biotek Sinergy H1).

### 2.10 Cell survival evaluation

PC3 or DU145 cells were seeded in 24‐well plates (1.5–3 × 10^4^ cells per well). Cells were subjected to specific treatments, as detailed in the figure legends. At the end point of the experiments, cells were detached with Trypsin–EDTA solution (Sigma‐Aldrich, #T4049), resuspended in DMEM supplemented with 10% FBS, 2 mm l‐glutamine, and 1% penicillin/streptomycin, and counted. Cell counts were performed using a 10× objective, according to standard procedure.

### 2.11 Lipid peroxidation analysis

PC3 cells were seeded in six‐well plates (1.5–2 × 10^5^ cells per well) and treated for 24 h with serum‐free medium or 20 mm lactic acid before incubation with 2 μm RSL3 for 4 h. Cells were then stained with 5 μm C11‐BODIPY^581/591^ (Thermo Fisher Scientific, #D3861) in Hank's Balanced Salt Solution for 15 min at 37 °C. Cells were collected after incubation with accutase, washed in PBS, and resuspended in PBS with 0.5% FBS. Live cells were analyzed by flow cytometry using FACS Canto II (BD Biosciences, Franklin Lakes, NJ, USA). Oxidation of the probe resulted in a shift of fluorescence from 590 to 510 nm, which was used for the analysis. 1 × 10^4^ cells were analyzed to measure the mean fluorescence intensity (MFI). Alternatively, PC3 cells were incubated for 24 h with serum‐free medium or CAF‐CM before treatment with RSL3. The ratio between green (λ_excitation_ = 485; λ_emission_ = 520) and red (λ_excitation_ = 560; λ_emission_ = 595) fluorescence of the probe C11‐BODIPY^581/591^, measured with the fluorometer Biotek Sinergy H1, was normalized on cell number.

### 2.12 RNAi transfection

PC3 cells at 70% confluence were transfected with siRNA targeting CA IX (15 nm) and CA XII (20 nm) or the negative control (siCTR, 35 nm) (Horizon Discovery Dharmacon, Lafayette, CO, USA; ON‐TARGET plus Human CA9 (768) siRNA‐SMARTpool #L‐005244‐00‐0005; ON‐TARGET plus Human CA12 (771) siRNA‐SMARTpool #L‐003634‐00‐0005; ON‐TARGET plus Non‐targeting Pool, #D‐001810‐10‐05) using Opti‐MEM (GIBCO‐Thermo Fisher Scientific, Waltham, MA, USA, #31985062) and Lipofectamine RNAiMAX Reagent (Thermo Fisher Scientific, #13778‐150), according to the manufacturer's instructions. Both silencing efficacy and functional assays were evaluated 72 h after transfection.

### 2.13 RNA extraction and quantitative real‐time PCR (qRT‐PCR) analysis

Total RNA was extracted using the RNAeasy Plus Mini Kit (Qiagen, Hilden, Germany, #74134) and quantified by NanoDrop One (Thermo Fisher Scientific). cDNA was synthesized from 500 to 1000 ng of total extracted RNA using the iScript cDNA Synthesis Kit (BioRad, #1725035) and the T100 ThermalCycler (BioRad), according to the manufacturer's instructions. qRT‐PCR was performed to quantify mRNA levels of selected targets by CFX96 Touch Real‐Time PCR Detection System (BioRad) using the TaqMan Universal PCR Master Mix (Applied Biosystem‐Thermo Fisher Scientific, Waltham, MA, USA, #4440040), according to manufacturer's instructions. The following probes were used: Carbonic Anhydrase 9 (CA9, Life Technologies‐Thermo Fisher Scientific, Carlsbad, CA, USA, #Hs00154208); Carbonic Anhydrase 12 (CA12, Life Technologies, #Hs01080909). Data are reported as relative quantity with respect to reference samples determined with the $2^{-\Delta \Delta C_{t}}$ method by cfx maestro software (BioRad). Data were normalized on TATA‐Box Binding Protein (TBP, Life Technologies, #Hs00427620) or Hypoxanthine Phosphoribosyltransferase 1 (HPRT1, Life Technologies, #Hs02800695).

### 2.14 Extracellular pH measurements

CAFs were treated for 48 h with serum‐free medium supplemented with 2 μm carbonic anhydrase inhibitors (SLC‐0111; CAI#1; CAI#2). Then, extracellular pH was measured in the extracellular media using the micropH combination electrode (Sigma‐Aldrich, #Z113425).

### 2.15 Lactate quantification

CAFs were incubated for 48 h with serum‐free medium supplemented with 2 μm carbonic anhydrase inhibitors (SLC‐0111; CAI#1; CAI#2). Extracellular medium was then collected and lactate secreted by CAFs was quantified using the L‐Lactic Acid Assay Kit (Megazyme, Bray, Wicklow, Ireland, #K‐LATE), according to the manufacturer's instructions.

### 2.16 Lactate uptake assay

To analyze the incorporation of exogenous lactic acid, CAFs and PC3 cells were incubated with 0.1 μCi·mL^−1^ lactic acid sodium salt L‐[^14^C(U)] (Perkin Elmer, Waltham, MA, USA, #NEC599050UC) in uptake buffer (140 mm NaCl, 20 mm HEPES/Na, 2.5 mm MgSO_4_, 1 mm CaCl_2_, 5 mm KCl, pH 7.4) for 15 min at 37 °C. Then, cells were washed in ice‐cold PBS and lysed with ice‐cold 0.1 m NaOH. Cell lysates were transferred to a scintillation vial and incorporated radioactivity was measured on a liquid scintillation counter (Tris‐Carb 2800TR, Perkin Elmer). The radioactivity measurements were normalized on protein content.

### 2.17 Statistical analysis

Statistical analyses were performed with graphpad prism version 10.3.1 for Windows (GraphPad Software, Boston, MA, USA, www.graphpad.com) on n biological independent replicates each performed with technical replicates (the number of biological and technical replicates are specified in the figure legends). Data are reported as mean ± standard error of the mean (SEM). Mathematical outliers were identified with Grubb's Test (α = 0.05) and removed from the analysis. Two‐tailed unpaired Student's *t*‐test or Mann–Whitney test was used to compare two groups. One‐way or Two‐way Analysis of Variance (ANOVA) was used when comparing multiple groups, applying specific post‐testing analysis (Dunnett, Tukey, Sidak) for individual comparisons with a confidence interval of 95%. For the correlation of IHC antibody staining on tumor tissues, a contingency table was analyzed using the Fisher's exact test. *P* value < 0.05 was considered statistically significant (ns, not significant; *P < 0.05; **P < 0.01; ***P < 0.001; ****P < 0.0001).

## 3 Results

### 3.1 GPX4 expression associates with aggressive features in prostate carcinoma and is upregulated upon lactic acid exposure

**Fig. 1:**
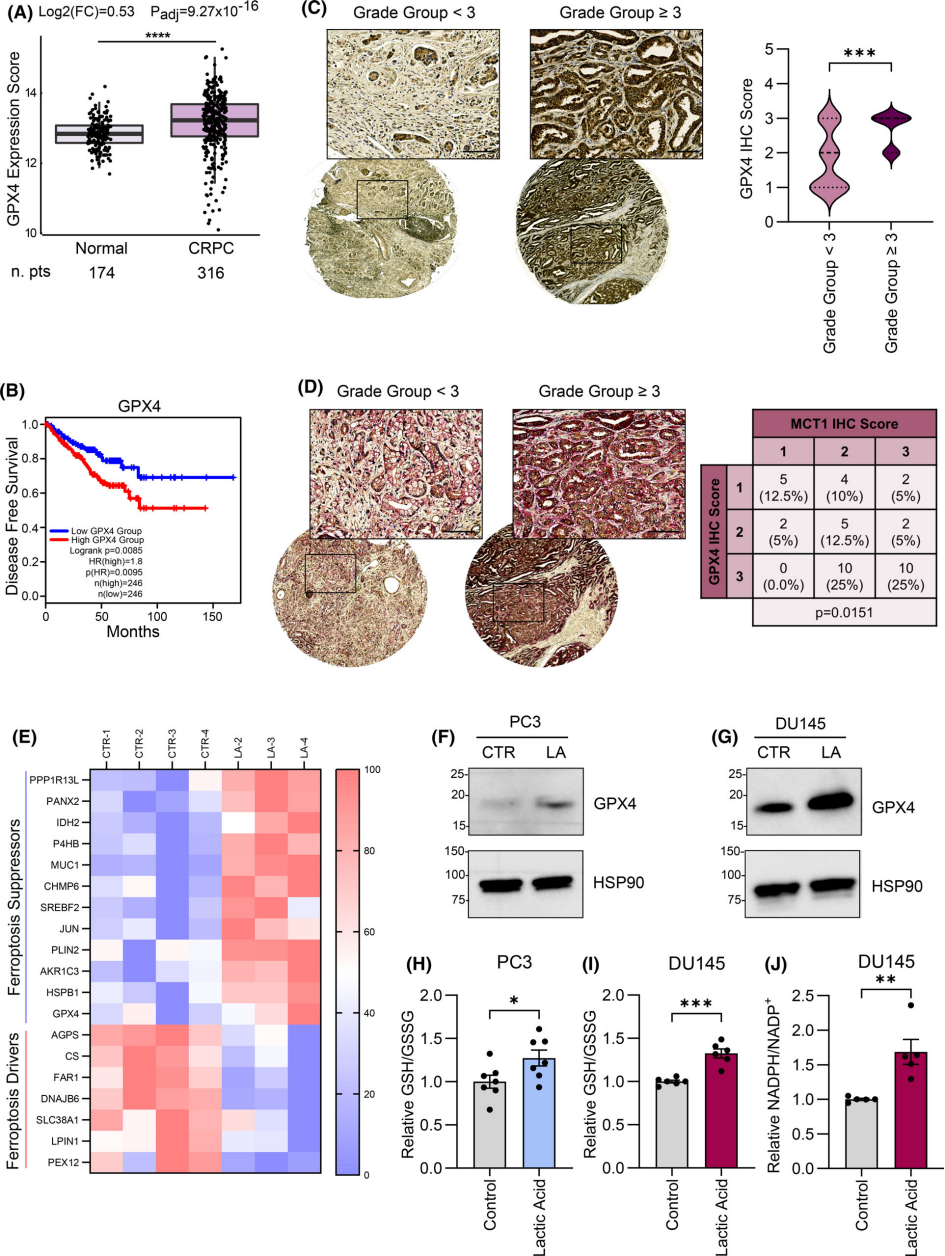
GPX4 expression positively associates with PCa progression. (A) GPX4 expression levels in CRPC patients vs. normal samples. Fold change (Log2(FC)) and adjusted *P* value (*P*adj) are reported. (B) Kaplan–Meier analysis of disease free survival in the PRAD Cohort (*n* = 492) stratified by GPX4 mRNA levels. HR, hazard ratio (95% confidence interval, 50–50% median cutoff). (C) GPX4 protein expression in tissue cores from a PCa TMA grouped by Grade Group (low Grade Group: Grade Group < 3; high Grade Group: Grade Group ≥ 3). Images of representative tissue cores (on the left, Grade Group = 2, on the right, Grade Group = 3) are reported (magnification 5× for the entire tissue core and 20× for the selected area; scale bar = 40 μm). On the right, violin plot of GPX4 histoscores (H‐score 1–3) in tissue cores (*n* = 45) of a PCa TMA grouped by low or high Grade Group. (D) IHC co‐staining for MCT1 (Fast Red chromogenic substrate) and GPX4 (DAB chromogenic substrate) in the PCa TMA cores. Representative images of high (Grade Group = 3) or low (Grade Group = 1) Grade Group tissue cores are reported (magnification 5× for the entire tissue core and 20× for the selected area; scale bar = 40 μm). On the right, co‐expression analysis of MCT1 and GPX4 in the PCa TMA. For each core of the TMA stained for MCT1 or GPX4, protein expression was quantified by H‐score (H‐score 1–3, *n* = 40). (E) Heatmap showing the vst‐normalized expression of ferroptosis‐related genes up‐ or downregulated in DU145 cells exposed to serum‐free medium (CTR1‐4) or 20 mm lactic acid (LA2‐4) for 48 h. (F, G) GPX4 protein levels evaluated by western blot analysis on total protein lysates from PC3 (F) and DU145 (G) cells treated with serum‐free medium (CTR) or 20 mm lactic acid (LA) for 48 h. HSP90 was used as loading control. Representative images of at least three independent experiments are reported. (H, I) GSH/GSSG ratio in PC3 (H) and DU145 (I) cells incubated with serum‐free medium (Control) or 20 mm lactic acid for 48 h. Relative GSH/GSSG ratio is reported using serum‐free medium‐treated cells as comparator (*n* = 4 biologically independent replicates, each performed in single or duplicate technical replicates). (J) NADPH/NADP^+^ ratio in DU145 cells incubated with serum‐free medium (Control) or 20 mm lactic acid for 48 h. Relative NADPH/NADP^+^ ratio is reported using serum‐free medium‐treated cells as comparator (*n* = 3 biologically independent replicates, each performed in single or duplicate technical replicates). In (H–J) data are reported as mean ± SEM of n biologically independent replicates, each performed with the indicated technical replicates. Statistical analyses were performed using the Wald test from deseq package (A), two‐tailed Mann–Whitney test (C), Fisher's exact test (D), and two‐tailed unpaired Student's *t*‐test (H–J). *P < 0.05; **P < 0.01; ***P < 0.001; ****P < 0.0001.

Among the various mechanisms governing ferroptosis sensitivity in cancer cells, the solute carrier family 7 member 11 (SLC7A11)—reduced glutathione (GSH)—GPX4 axis plays a fundamental role [35]. Specifically, SLC7A11, the light chain of the system $x_{c}^{-}$, mediates the import of exogenous cystine, which is a crucial building block for GSH synthesis, and its overexpression protects cells from lipid peroxidation‐induced death [34]. Besides, GPX4 antagonizes ferroptosis by reducing toxic PUFA‐phospholipid hydroperoxides into their corresponding nonreactive alcohols, using GSH as a cofactor [51]. In this context, *in silico* data analysis of a cohort of PCa patient samples [47] revealed that GPX4 expression is more elevated in castration‐resistant prostate cancer (CRPC) tissues compared to normal prostate (Fig. 1A). Accordingly, GPX4‐high expressing PCa patients from PRAD‐TCGA cohort display a significant shorter disease‐free survival than those that are GPX4‐low expressing (Fig. 1B). To confirm the clinical relevance of GPX4 in PCa, we performed an immunohistochemistry (IHC) analysis of a tissue microarray (TMA) composed of tumor tissue cores from patients affected by PCa at different stages. Remarkably, GPX4 protein levels were higher in tissues from patients with high Grade Group PCa (Grade Group ≥ 3), compared to the low Grade Group tissues (Grade Group < 3) (Fig. 1C). These results demonstrate that enhanced GPX4 levels correlate with a more aggressive phenotype and worse prognosis in PCa and are in agreement with previous evidence in different tumor models [52, 53, 54]. Recent preclinical findings underlined that PCa cells resistant to androgen deprivation therapy are vulnerable to FINs [40]. However, the efficacy of FINs might depend on non‐cell autonomous mechanisms, such as TME‐enriched metabolites [32]. Among the different nutrients, lactic acid is particularly abundant in the tumor milieu, being produced by various TME cellular sources, including tumor, stromal and immune cells [8, 9, 55]. This evidence led us to investigate the role of microenvironmental lactic acid in the modulation of ferroptosis sensitivity in PCa. A recent study performed using real‐time imaging on humans underscored that lactate levels are increased in prostate tumors with high Gleason grade and lactate‐enriched regions are characterized by elevated MCT1 expression [56]. Accordingly, we formerly highlighted that MCT1 protein expression positively correlates with the Grade Group of PCa patients, and is thus associated with increased aggressive traits [16]. We integrated these results by performing an IHC TMA co‐staining of MCT1 and GPX4, and we observed that GPX4 expression levels were higher in tissues with increased MCT1 expression (Fig. 1D), suggesting that GPX4 might play a role in lactate‐enriched (i.e., MCT1 high) PCa tissues. As lactic acid has been previously found to sustain a wide transcriptional reprogramming [16], we intersected normalized and significantly modulated genes from RNA‐seq analysis performed on lactic acid‐treated DU145 cells [16] with a list of ferroptosis‐associated genes, based on FerrDb database [47] (Fig. 1E; Table S1). Notably, lactic acid exposure positively and negatively regulated the expression of many ferroptosis suppressor and driver genes, respectively. Among them, GPX4 was upregulated following lactic acid treatment (Fig. 1E; Table S1). Accordingly, the evaluation of GPX4 protein levels in two different PCa cell lines (PC3 and DU145) treated for 48 h with 20 mm lactic acid (i.e., a concentration of the nutrient that resembles the one found in *in vivo* tumor tissues and that takes into account the contribution of the heterogenous cell populations within the prostate TME [7, 17, 57, 58]) confirmed its overexpression upon lactic acid exposure (Fig. 1F,G and Fig. S1A,B), hence suggesting that microenvironmental lactic acid could mediate ferroptosis resistance in PCa. GPX4 activity is regulated by GSH levels, acting as a cofactor of the peroxidase [51]. GSH is in turn regenerated from oxidized glutathione (GSSG) through the activity of glutathione reductase, that requires NADPH as cofactor for the catalysis. In accordance, we found that exogenous lactic acid induced an unbalance in GSH/GSSG ratio (Fig. 1H,I) and NADPH/NADP^+^ ratio (Fig. 1J) in PCa cells, indicative of its protective role against oxidative stress, potentially contributing to ferroptosis resistance. Altogether, the upregulation of GPX4 protein levels and the alteration of redox balance following lactic acid exposure are both supportive of lactic acid‐mediated ferroptosis resistance in PCa.

### 3.2 CAF‐secreted lactic acid supports the acquisition of a ferroptosis‐resistant phenotype in PCa cells

**Fig. 2:**
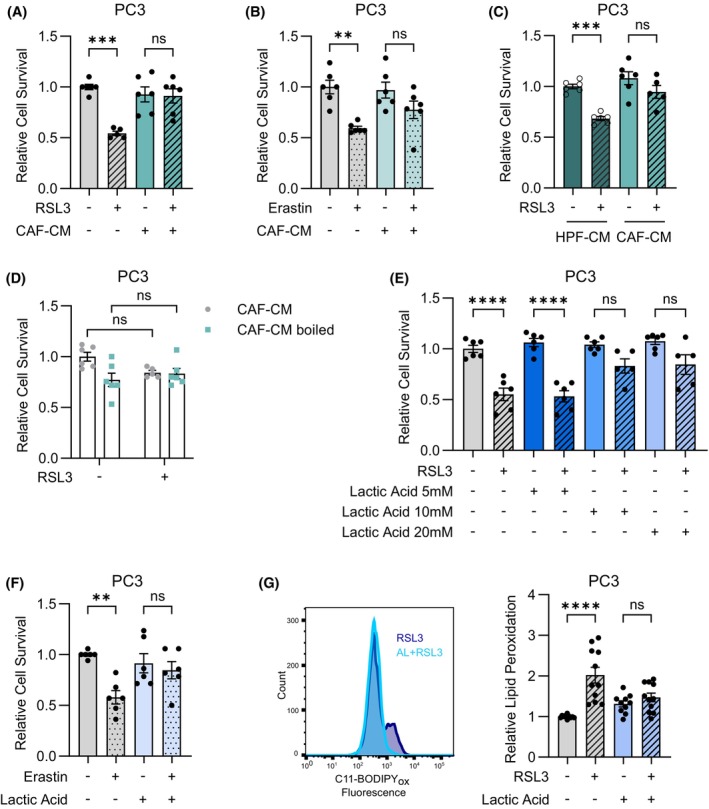
Stromal lactic acid protects PCa cells from RSL3 and Erastin‐induced ferroptosis. (A, B) PC3 cells were incubated with serum‐free medium or CAF‐CM for 24 h and then treated for additional 24 h with 1 μm RSL3 (A) or 10 μm Erastin (B) before evaluation of cell viability. Relative cell survival is reported using serum‐free medium‐treated cells as comparator (A, *n* = 4 biologically independent replicates, each performed in single or duplicate technical replicates; B, *n* = 3 biologically independent replicates, each performed in duplicate technical replicates). (C) PC3 cells were incubated with HPF‐CM or CAF‐CM for 24 h, before treatment with 1 μm RSL3 for further 24 h. Cell viability was then evaluated. Relative cells survival is reported using HPF‐CM‐treated cells as comparator (*n* = 3 biologically independent experiments, each performed in single or duplicate technical replicates). (D) CM was isolated from CAFs and then boiled (CAF‐CM boiled) or used as native CM. PC3 cells were then exposed to boiled or native CAF‐CM for 24 h before treatment with 1 μm RSL3 for additional 24 h. Then, cell viability was evaluated. Relative cell survival is reported using native CAF‐CM‐treated cells as comparator (*n* = 3 biologically independent replicates, each performed in duplicate technical replicates). (E) PC3 cells were incubated with serum‐free medium (0 mm) or with increasing doses of exogenous lactic acid (5, 10, 20 mm) for 24 h. Then, cancer cells were treated with 1 μm RSL3 for further 24 h before evaluation of cell viability. Relative cell survival is reported using serum‐free medium‐treated cells as comparator (*n* = 3 biologically independent replicates, each performed in single or duplicate technical replicates). (F) PC3 cells were treated with serum‐free medium or 20 mm lactic acid for 24 h and then incubated with 10 μm Erastin for additional 24 h before evaluation of cell viability. Relative cell survival is reported using serum‐free medium‐treated cells as comparator (*n* = 4 biologically independent replicates, each performed in single or duplicate technical replicates). (G) PC3 cells were treated with serum‐free medium or 20 mm lactic acid for 24 h and then incubated with 2 μm RSL3 for 4 h. Lipid peroxidation levels were measured by staining cells with 5 μm C11‐BODIPY^(581/591)^ for 15 min at 37 °C and then performing cytofluorimetric analysis to evaluate the mean fluorescence intensity (MFI) of the oxidized probe. Relative lipid peroxidation is reported using serum‐free medium‐treated cells as comparator (*n* = 5 biologically independent replicates, each performed in duplicate technical replicates). A representative flow chart is reported. In (A–G) data are reported as mean ± SEM of n biological independent experiments, each performed with the indicated technical replicates. Statistical analyses were performed using one‐way ANOVA followed by Tukey's multiple comparisons test (A–C, E–G) or two‐way ANOVA followed by Sidak's multiple comparisons test (D). ns, not significant; **P < 0.01; ***P < 0.001; ****P < 0.0001.

Given the role of exogenous lactic acid in upregulating GPX4 expression levels, we explored whether this metabolite could protect PCa cells from cell death promoted by FINs (e.g., RSL3 and Erastin). CAFs are the most abundant cell population within the tumor reactive stroma and represent an important cellular source of lactic acid in PCa [4, 15, 17, 59]. Thus, we first assessed the role of CAFs in modulating ferroptosis sensitivity of PCa cells, by incubating PC3 and DU145 cells with serum‐free medium or conditioned media isolated from CAFs (CAF‐CM) for 24 h before treatment with RSL3 and Erastin for additional 24 h. Specifically, RSL3 acts as a GPX4 inhibitor [51, 60], while Erastin triggers ferroptosis through system $x_{c}^{-}$ inhibition [34, 61]. Interestingly, RSL3 and Erastin induced a significant reduction in cancer cell survival. However, the preconditioning with CAF‐CM almost completely prevented the RSL3‐ and Erastin‐induced decrease in cancer cell viability (Fig. 2A,B and Fig. S1C,D). In agreement with a CAF‐supported ferroptosis‐resistant phenotype, exposure to CAF‐CM also led to an increase in GPX4 protein expression levels in PCa cells (Fig. S1E). We then evaluated whether the effects of CAF‐CM on the modulation of ferroptosis sensitivity of PCa cells were driven by the specific environment generated following the activation of stromal fibroblasts within the TME. We thus assessed cell survival of PCa cells incubated with CM from either healthy prostate fibroblasts (HPFs) or CAFs for 24 h, and then treated for further 24 h with RSL3. Interestingly, whereas incubation with CAF‐CM prevented RSL3‐induced cell death in PCa cells, CM isolated from HPFs did not confer protection from ferroptosis to tumor cells (Fig. 2C). To discriminate the specific role of CAF‐CM secreted metabolites or protein factors in mediating the observed CAF‐CM‐induced ferroptosis protection, we investigated the effect of incubating PCa cells with CM that had been deprived of all heat‐labile proteins by heat shock, without affecting their metabolite composition. Of note, the protein‐deprived CM retained the protective effect toward RSL3‐induced cell death observed upon untreated CM incubation (Fig. 2D), further reinforcing our hypothesis that specific soluble non‐protein mediators (i.e., metabolites) are responsible for the CM‐induced ferroptosis resistance. Thus, to verify the specific role of lactic acid in the regulation of cell death promoted by FINs, PCa cells were incubated with increasing concentrations of exogenous lactic acid, in the range 0–20 mm, for 24 h, before treatment with RSL3 for additional 24 h. Indeed, lactic acid concentration rises from 1.5 to 3 mm under physiological conditions, as evaluated in blood and tissues from healthy individuals, to 10–40 mm in inflammatory pathologies and tumor tissues [17, 62, 63, 64]. We previously demonstrated that the levels of CAF‐secreted lactic acid in *in vitro* settings ranged around 10 mm, while its levels in the extracellular media of HPFs resulted to be approximately 3–4 mm [17]. Interestingly, we found that 5 mm lactic acid did not protect PCa cells from ferroptosis. Conversely, higher concentrations of lactic acid (10 and 20 mm) similarly prevented RSL3‐induced decrease in cell survival (Fig. 2E and Fig. S1F). This result is in line with the evidence that CM isolated from HPFs, secreting lower doses of lactic acid in the extracellular media, did not prevent ferroptosis in PCa cells, while PCa cells exposed to lactic acid‐enriched CAF‐CM displayed resistance to RSL3‐induced cell death (Fig. 2C). We then assessed cell viability of PCa cells treated for 24 h with 20 mm lactic acid (i.e., a concentration of the nutrient that better reproduces the metabolic heterogeneity within the tumor mass [7, 17]) and for further 24 h with Erastin. Remarkably, lactic acid also negatively affected the sensitivity of PCa cells to Erastin‐induced ferroptosis (Fig. 2F and Fig. S1G). Overall, comparably to CAF‐CM, lactic acid protected PCa cells from cell death induced by RSL3 and Erastin. Accumulation of toxic lipid peroxides at cell membranes mediated by high intracellular reactive oxygen species (ROS) levels is the main driver of ferroptosis [65]. Hence, we measured the oxidation status of C11‐BODIPY^581/591^, a fluorescent peroxidation indicator shifting from a reduced to an oxidized form in presence of lipid ROS [34]. Remarkably, exogenous lactic acid avoided the shift of the fluorescent probe to its oxidized form, which was instead observed following 4 h of incubation with RSL3 (Fig. 2G), indicating that lactic acid exposure prevents the accumulation of lipid peroxides in PCa cells. Analogously, CAF‐CM impaired the increase in lipid peroxidation in cancer cells exposed to RSL3 (Fig. S1H). Taken together, these results underscore that CAF‐secreted lactic acid acts as a key driver of ferroptosis resistance in PCa.

### 3.3 Interfering with MCT1 activity sensitizes lactic acid‐exposed PCa cells to RSL3‐induced ferroptosis

**Fig. 3:**
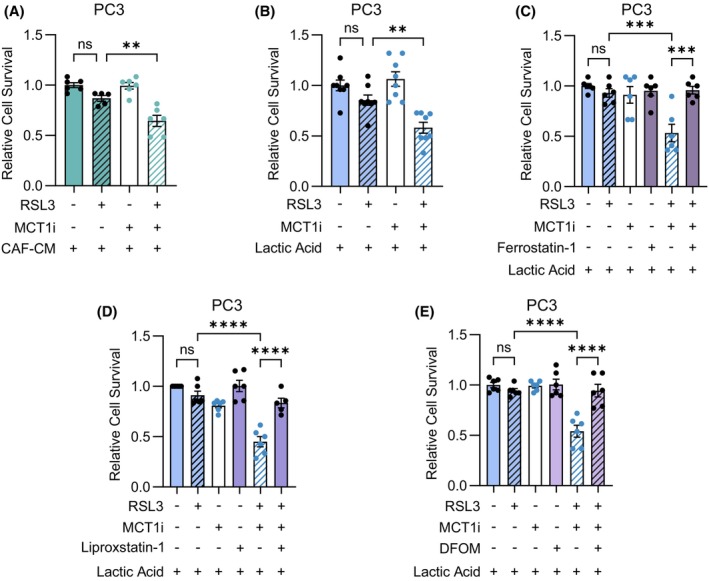
MCT1 targeting prevents lactic acid‐mediated resistance to ferroptosis in PCa cells. (A) PC3 cells were exposed to CAF‐CM in the presence or not of 40 μm MCT1 inhibitor (MCT1i) AR‐C155858. After 24 h, 1 μm RSL3 was added to the cell cultures and after additional 24 h cell viability was evaluated (*n* = 3 biologically independent replicates, each performed in duplicate technical replicates). Relative cell survival is reported using CAF‐CM‐treated cells as comparator. (B) PC3 cells were incubated with 20 mm lactic acid in the presence or not of 40 μm MCT1i AR‐C155858 for 24 h. Then, cells were treated with 1 μm RSL3 and after additional 24 h cell viability was evaluated (*n* = 3 biologically independent replicates, each performed in duplicate or triplicate technical replicates). (C–E) PC3 cells were exposed for 24 h to 20 mm lactic acid in the presence or not of 40 μm MCT1i AR‐C155858. Then, cells were pre‐treated for 2 h with 1 μm Ferrostatin‐1 (C) or 1 μm Liproxstatin‐1 (D) or 10 μm DFOM (E) before incubation with 1 μm RSL3 for 24 h. Then, cell viability was assessed (*n* = 3 biologically independent replicates, each performed in duplicate technical replicates). In (B–E) relative cell survival is reported using lactic acid‐treated cells as comparator. In (A–E) data are reported as mean ± SEM of *n* biological independent experiments, each performed with the indicated technical replicates. Statistical analyses were performed using one‐way ANOVA followed by Dunnett's multiple comparisons test (A, B) or Tukey's multiple comparisons test (C–E). ns, not significant; **P < 0.01; ***P < 0.001; ****P < 0.0001.

MCT1‐4 act as passive lactate/H^+^ symporters, mediating the bidirectional cotransport of lactate and protons according to their concentration gradients. In several solid tumors, including PCa, oxidative cells face MCT1‐mediated uptake of exogenous lactic acid that is secreted in the extracellular environment by MCT4‐expressing glycolytic cells [19]. To further corroborate the role of CAF‐secreted lactic acid in providing ferroptosis protection, we interfered with its MCT1‐dependent uptake in PCa cells using the compound AR‐C155858 (MCT1i). Of note, MCT1 inhibition prevented the protective effect elicited by CAF‐CM or lactic acid in RSL3‐exposed PCa cells (Fig. 3A,B and Fig. S2A), confirming that stromal lactic acid intake by cancer cells results in ferroptosis resistance. Additionally, the decrease in cell survival promoted by cotreatment of PCa cells with MCT1i and RSL3 upon exposure to lactic acid was completely reversed by the ferroptosis inhibitors ferrostatin‐1, liproxstatin‐1, and deferoxamine mesylate salt (DFOM) [34, 66, 67, 68] (Fig. 3C–E and Fig. S2B–D). It is noteworthy that upon induction of ferroptosis, following treatment with RSL3, PCa cells increased the incorporation of ^14^C‐radiolabeled lactate (Fig. S2E). This might represent a ‘defensive mechanism’ to resist RSL3‐induced cell death, further corroborating the role of lactic acid as a negative regulator of ferroptosis in PCa. Overall, these findings support the hypothesis that exogenous lactic acid specifically mediates resistance to ferroptosis, and interfering with stroma‐derived lactic acid transport across cell membranes via MCT1 inhibition sensitizes PCa cells to this form of cell death.

### 3.4 The anti‐ferroptotic effect of stromal lactic acid is prevented by CA IX/XII targeting in prostate CAFs

**Fig. 4:**
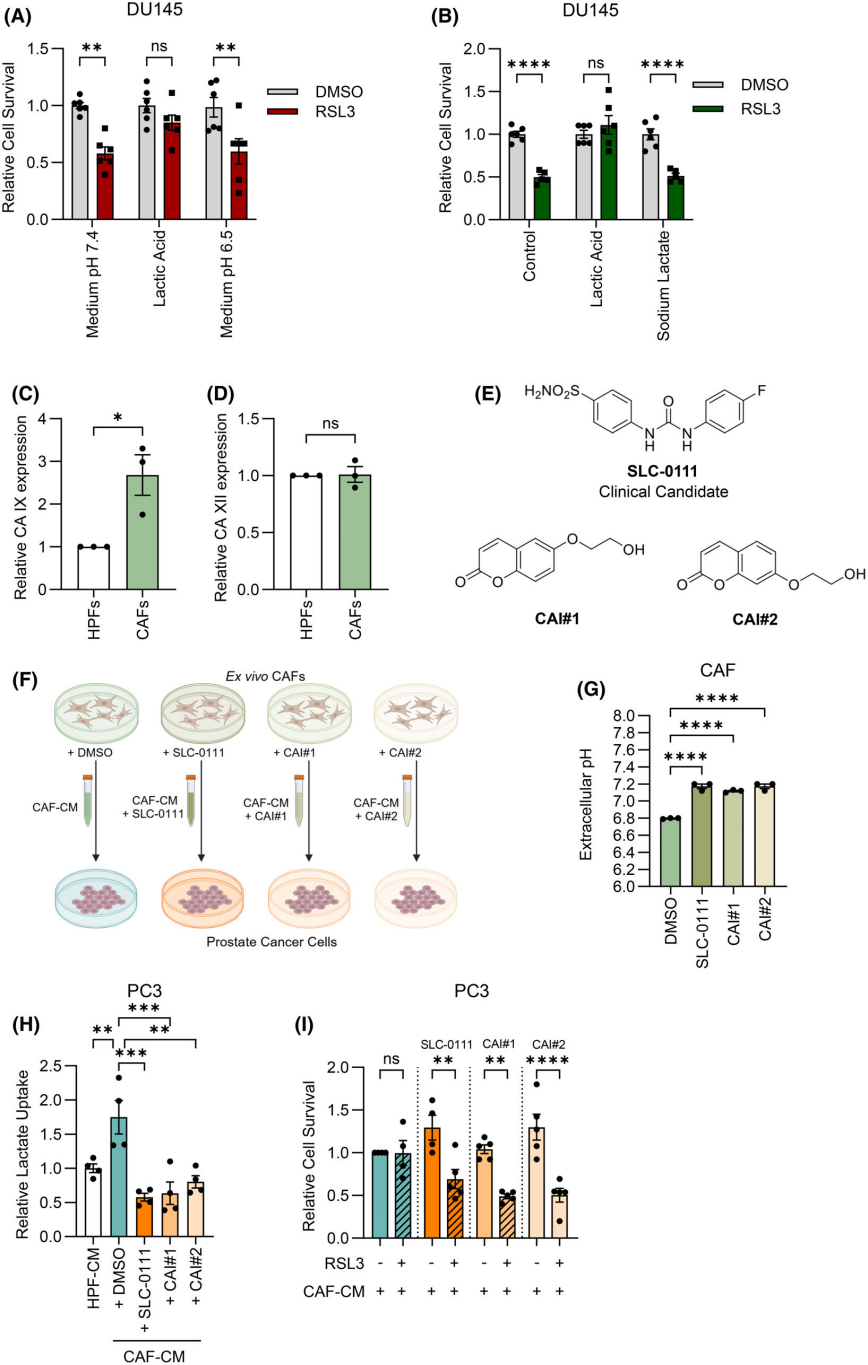
Targeting of CA IX and CA XII in stromal cells sensitizes PCa cells to ferroptosis. (A) Cell survival was evaluated in DU145 cells treated for 24 h with serum‐free medium (pH 7.4), 20 mm lactic acid, or acidic medium (pH 6.5) and for additional 24 h with 1 μm RSL3 (*n* = 3 biologically independent replicates, each performed in duplicate technical replicates). Data are reported as relative cell survival of RSL3‐treated vs. untreated conditions for each cell treatment. (B) Cell survival was evaluated in DU145 cells treated for 24 h with serum‐free medium (Control), 20 mm lactic acid, or 20 mm sodium lactate and for additional 24 h with 1 μm RSL3 (*n* = 3 biologically independent replicates, each performed in duplicate technical replicates). Data are reported as relative cell survival of RSL3‐treated vs. untreated conditions for each cell treatment. (C) mRNA levels of CA IX in HPFs (*n* = 3) and CAFs (*n* = 3) evaluated by quantitative RT‐PCR. Relative expression is reported using HPFs as comparator. (D) mRNA levels of CA XII in HPFs (*n* = 3) and CAFs (*n* = 3) evaluated by quantitative RT‐PCR. Relative expression is reported using HPFs as comparator. (E) Chemical structures of CA IX/XII inhibitors utilized for cell treatments. (F) Schematic representation of CAF and PCa cell treatments with CA IX/XII inhibitors. Image was created using Biorender.com. (G) Extracellular pH values measured in culture media of untreated CAFs (DMSO) or CAFs exposed for 48 h to 2 μm CA IX/XII inhibitors (SLC‐0111, CAI#1, CAI#2) (*n* = 3 biologically independent replicates). (H) CAFs were treated for 48 h with serum‐free medium in the presence or not of 2 μm CA IX/XII inhibitors (SLC‐0111, CAI#1, CAI#2). Untreated HPFs were used as control. Then, PC3 cells were incubated for 48 h with CM isolated from HPFs, untreated CAFs and CA IX/XII inhibitor‐treated CAFs. Tumor cells were exposed to ^14^C‐lactate‐containing medium and incorporation of exogenous ^14^C‐lactate was measured and normalized on protein content (*n* = 2 biologically independent replicates, each performed in duplicate technical replicates). (I) CAFs were treated for 48 h with serum‐free medium in the presence or not of 2 μm CA IX/XII inhibitors (SLC‐0111, CAI#1, CAI#2). Then, PC3 cells were incubated for 24 h with CM isolated from untreated CAFs and CA IX/XII inhibitor‐treated CAFs before incubation for additional 24 h with 1 μm RSL3. Cell survival was then evaluated. Relative cell survival is reported using CAF‐CM‐treated cells as comparator (*n* = 4 biologically independent replicates, each performed in single or duplicate technical replicates). In (A–D) and (G–I) data are reported as mean ± SEM of n biological independent experiments, each performed with the indicated technical replicates. Statistical analyses were performed using two‐way ANOVA followed by Sidak's multiple comparisons test (A, B), two‐tailed unpaired Student's *t*‐test (C, D) or one‐way ANOVA followed by Dunnett's multiple comparisons test (G, H) or Tukey's multiple comparisons test (I). ns, not significant; *P < 0.05; **P < 0.01; ***P < 0.001; ****P < 0.0001.

Highly glycolytic prostate CAFs are characterized by increased lactic acid secretion and reduced extracellular pH [4, 30]. Indeed, lactate/H^+^ extrusion by glycolytic cells is responsible for extracellular milieu acidification [6, 69]. Hence, we examined whether the stromal lactic acid‐driven regulation of ferroptosis sensitivity in PCa cells could be attributed to: (a) the acidity as a consequence of lactic acid export *per se*; (b) the nutrient lactate *per se*; and (c) a combination of extracellular acidification and microenvironmental lactate. We thus initially treated DU145 cells with either serum‐free medium (pH 7.4), lactic acid, or acidic medium (pH 6.5), before incubation with RSL3. Of note, while exposure to exogenous lactic acid prevented the RSL3‐induced reduction of cell survival, the same effect was not observed under acidic conditions, with a decrease of cell survival in the presence of RSL3 (Fig. 4A). DU145 cells were then incubated with sodium lactate or lactic acid and, interestingly, sodium lactate did not recapitulate the protective effect against RSL3‐induced ferroptosis, which was conversely pursued by lactic acid (Fig. 4B). Together, these results demonstrate that neither the acidic milieu nor the nutrient lactate *per se* are sufficient to protect from ferroptosis and suggest a combined role of extracellular acidosis and a lactate‐enriched environment in supporting ferroptosis resistance in PCa. The acidic TME is maintained by various adaptive mechanisms, including MCTs, Na^+^/H^+^ exchangers, H^+^/K^+^ ATPases, Na^+^/${\mathrm{HCO}}_{3}^{-}$ co‐transporters, and CAs [22]. In particular, extracellular‐facing CA IX and CA XII are important for ensuring acid diffusion across the extracellular space in tumors [25, 27, 28] and CA IX is crucial for CAF‐promoting PCa cell aggressiveness [30, 31]. In keeping, we evaluated if CA IX/XII targeting in stromal cells could regulate ferroptosis sensitivity of PCa cells through alterations of TME acidosis. We confirmed in freshly isolated primary fibroblasts that CAFs were characterized by increased CA IX expression levels, compared to HPFs (Fig. 4C). CA XII was also expressed by CAFs, although its levels were not upregulated in CAFs with respect to HPFs (Fig. 4D). Prostate CAFs were treated with three different pharmacological CA IX/XII inhibitors, including the clinical candidate SLC‐0111, and two synthesized compounds, namely CAI#1 and CAI#2 (Fig. 4E,F). To this purpose, we confirmed that CA IX/XII inhibitors increased the extracellular pH of CAF‐CM (Fig. 4G), without altering the levels of secreted lactic acid (Fig. S3A). Then, to evaluate tumor‐stroma interplay under CA IX/XII targeting conditions, PCa cells were incubated with CM isolated from CAFs, previously treated with CA IX/XII inhibitors (Fig. 4F). We firstly assessed tumor cell ability to import stromal lactic acid by measuring the incorporation of ^14^C‐radiolabeled lactic acid. Interestingly, in the presence of CM from CA IX/XII‐inhibited CAFs, the upload of exogenous lactic acid by PCa cells was strongly reduced (Fig. 4H), suggesting that microenvironmental acidosis is potentially necessary to facilitate lactate/H^+^ transport across the plasma membrane in PCa cells. Then, we evaluated if stromal CA‐mediated perturbations of the acidic milieu also affect the ability of lactic acid‐experiencing cancer cells to resist ferroptosis. Remarkably, the ferroptosis protective effect of CAF‐CM in PCa cells was strongly abolished following CA IX/XII inhibition in stromal cells (Fig. 4I and Fig. S3B). Hence, these data indicate that targeting CAs facing the extracellular environment in stromal cells reduces microenvironmental acidosis, thereby impacting the co‐transport of lactate/H^+^ across the plasma membrane and consequently preventing stromal lactic acid‐mediated ferroptosis resistance in PCa cells.

### 3.5 Tumor‐expressed CA IX/XII are involved in the regulation of ferroptosis sensitivity upon lactic acid exposure

**Fig. 5:**
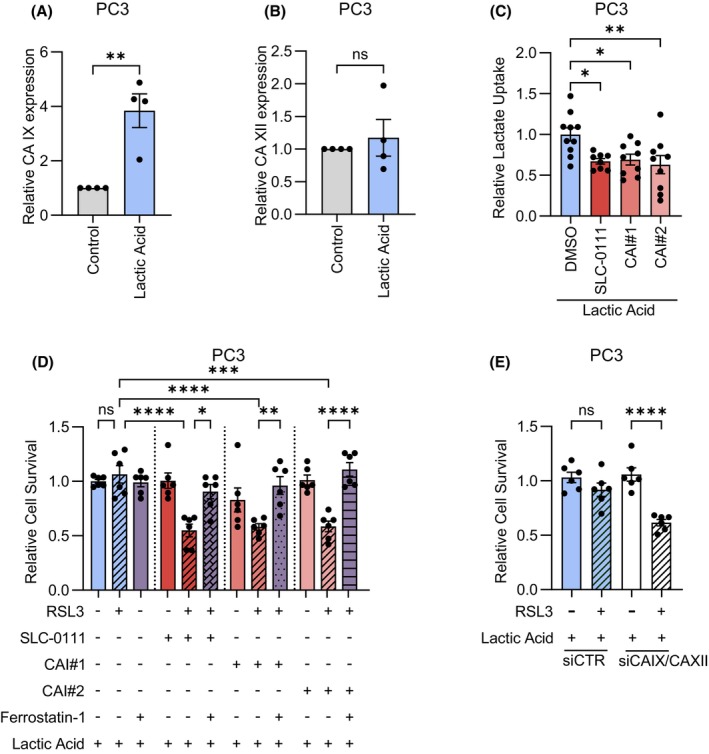
Targeting of CA IX and CA XII in lactic acid‐primed PCa cells affects ferroptosis sensitivity. (A, B) mRNA levels of CA IX (A) and CA XII (B) evaluated by qRT‐PCR in PC3 cells exposed for 48 h to serum‐free medium (Control) or 20 mm lactic acid (*n* = 4 biologically independent replicates). Relative expression is reported using control cells as comparator. (C) PC3 cells were treated for 48 h with 20 mm lactic acid in the presence or not of 2 μm CA IX/XII inhibitors (SLC‐0111, CAI#1, CAI#2). Then, the incorporation of exogenous ^14^C‐lactate was measured and normalized on protein content (*n* = 3 biologically independent replicates, each performed in at least triplicate technical replicates). Relative lactate uptake is reported using lactic acid‐treated cells as comparator. (D) PC3 cells were exposed for 24 h to 20 mm lactic acid in the presence or not of 2 μm CA IX/XII inhibitors (SLC‐0111, CAI#1, CAI#2). Then, cells were pre‐treated for 2 h with 1 μm Ferrostatin‐1 before incubation with 1 μm RSL3 for 24 h and cell viability was evaluated. Relative cell survival is reported using lactic acid‐treated cells as comparator (*n* = 3 biologically independent replicates, each performed in duplicate technical replicates). (E) PC3 cells were transiently silenced using non‐targeting control (siCTR) or CA IX and CA XII selective combo siRNA (siCA IX/XII). Then, cells were treated for 24 h with 20 mm lactic acid, followed by incubation for additional 24 h with 1 μm RSL3. Cell viability was then assessed (*n* = 4 biologically independent replicates, each performed in single or duplicate technical replicates). RSL3‐treated conditions were normalized on the corresponding siCTR or siCA IX/XII untreated conditions. In (A–E) data are reported as mean ± SEM of *n* biological independent experiments, each performed with the indicated technical replicates. Statistical analyses were performed using two‐tailed unpaired Student's *t*‐test (A, B) or one‐way ANOVA followed by Dunnett's multiple comparisons test (C) or Tukey's multiple comparisons test (D, E). ns, not significant. *P < 0.05; **P < 0.01; ***P < 0.001; ****P < 0.0001.

CA inhibitors act as systemic agents, targeting not only the stromal compartment but also tumor cells. Recent findings reported a role for tumor CA IX in ferroptosis regulation in breast cancer and malignant mesothelioma [70, 71]. We thus asked whether CA IX/XII inhibitors might modulate ferroptosis sensitivity also by targeting CAs on tumor cells themselves. Previous evidence dissecting the tumor‐stroma interplay in PCa underlined that upon exposure to CAF‐CM, PCa cells upregulate CA IX expression [30]. Interestingly, we observed that exogenous lactic acid supported the increase in CA IX expression in PCa cells, without affecting CA XII levels (Fig. 5A,B and Fig. S3C,D). Incubating PCa cells with SLC‐0111, CAI#1, and CAI#2 for 48 h reduced their ability to upload exogenous lactic acid (Fig. 5C), suggesting that CA IX/XII‐mediated control of extracellular pH homeostasis is important to facilitate the symport lactate/H^+^. In addition, analogously to what was observed with CA IX/XII targeting in the stromal compartment, inhibition of CA IX/XII in tumor cells sensitized lactic acid‐primed PCa cells to RSL3‐induced ferroptosis, while Ferrostatin‐1 blocked the reduction of cell survival (Fig. 5D and Fig. S3E). Hence, CA IX/XII inhibitors prevent lactic acid‐supported ferroptosis resistance by limiting lactic acid import in PCa cells. To confirm that the observed phenotypic effects on ferroptosis regulation were mediated by CA IX/XII targeting and not by off‐target events induced by the drugs, we performed a simultaneous silencing for both CA IX and CA XII isoform genes (Fig. S3F,G). Accordingly, we observed that, in the presence of exogenous lactic acid, control cells were refractory to RSL3‐induced survival drop. By contrast, CA IX/XII co‐silenced PCa cells were vulnerable to RSL3‐promoted cell death (Fig. 5E). Overall, these findings highlight that systemic administration of CA IX/XII inhibitors, beyond their effects on the stromal compartment, directly affects the ability of cancer cells to upload exogenous lactic acid, thereby acting as ferroptosis sensitizers.

### 3.6 CA IX and CA XII protein levels increase during PCa progression

**Fig. 6:**
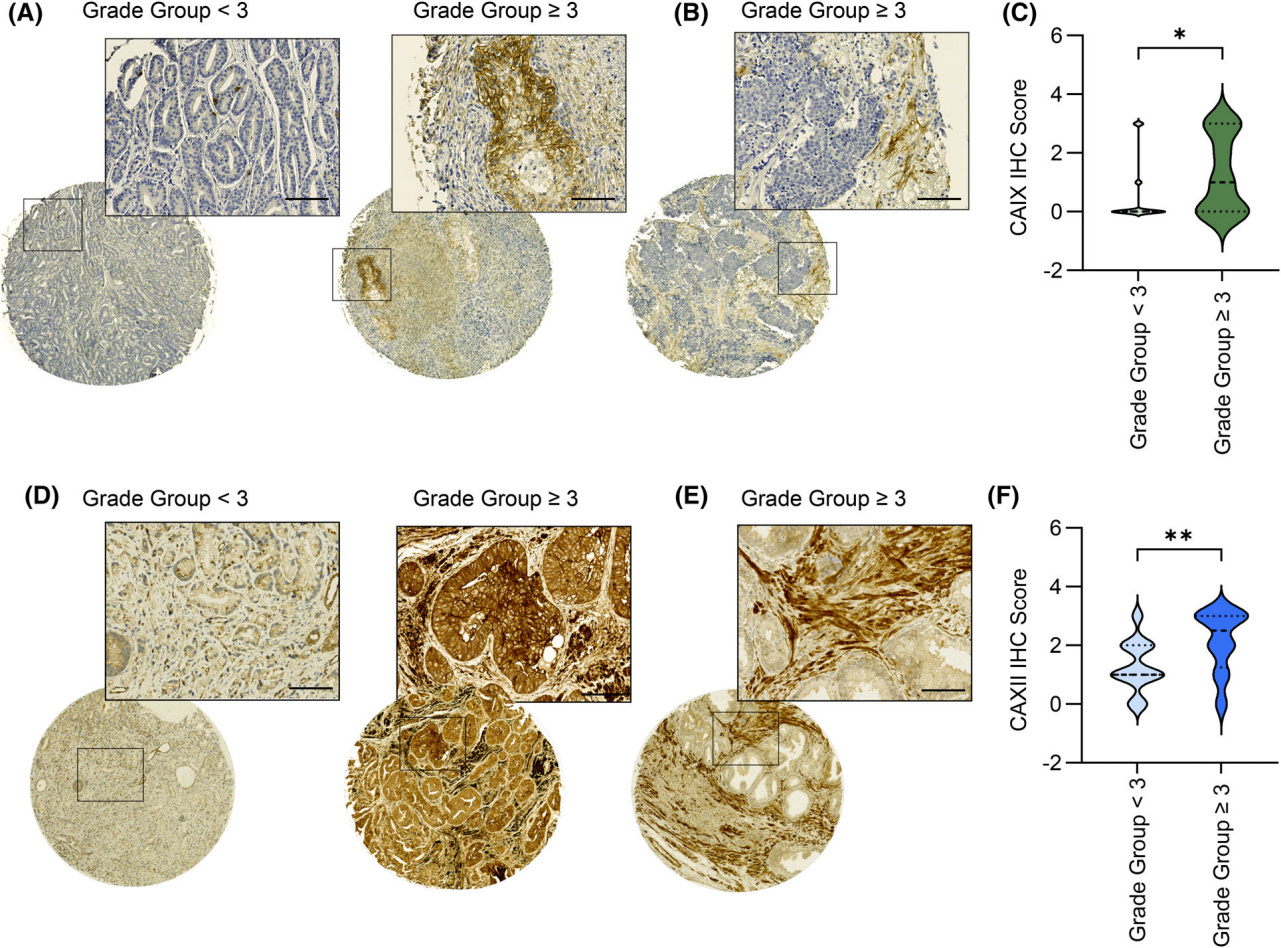
*Ex vivo* CA IX and CA XII expression correlate with an aggressive phenotype in prostate carcinoma. (A) CA IX protein expression in tissue cores from a PCa TMA grouped by Grade Group (low Grade Group: Grade Group < 3; high Grade Group: Grade Group ≥ 3). Images of representative tissue cores (on the left, Grade Group = 1; on the right, Grade Group = 5) are reported (magnification 5× for the entire tissue core and 20× for the selected area, scale bar = 40 μm). (B) Representative images of CA IX protein expression in the stromal compartment of tissue cores from the PCa TMA (Grade Group = 5; magnification 5× for the entire tissue core and 20× for the selected area, scale bar = 40 μm). (C) Violin plot of CA IX histoscores (H‐score 0–3) in tissue cores (*n* = 39) grouped by low or high Grade Group. (D) CA XII protein expression in tissue cores from a PCa TMA grouped by Grade Group. Images of representative tissue cores (on the left, Grade Group = 1; on the right, Grade Group = 5) are reported (magnification 5× for the entire tissue core and 20× for the selected area, scale bar = 40 μm). (E) Representative images of CA XII protein expression in the stromal compartment of tissue cores from the PCa TMA (Grade Group = 4; magnification 5× for the entire tissue core and 20× for the selected area, scale bar = 40 μm). (F) Violin plot of CA XII histoscores (H‐score 0–3) in tissue cores (*n* = 44) grouped by low or high Grade Group. Statistical analyses were performed using two‐tailed Mann–Whitney test. *P < 0.05, **P < 0.01.

To validate the clinical relevance of CA IX and CA XII expression during PCa progression, we screened the PCa TMA for CA IX and CA XII IHC staining. In line with previous evidence [72], CA IX protein levels were increased in tissue cores from high Grade Group patients, disclosing a positive association between CA IX expression and PCa progression (Fig. 6A–C). Analogously, CA XII protein expression was enhanced in more aggressive prostate tumors, while its staining was weaker in low Grade Group tumors, further indicating that CA XII expression positively correlates with Grade Group in prostate carcinoma (Fig. 6D–F). Although this isoform has not been previously investigated in PCa, we could hypothesize that CA XII might represent a prognostic factor for PCa progression. Interestingly, IHC analyses revealed that CA XII staining in PCa patient tissues was stronger than CA IX staining, suggesting that CA XII expression levels are higher when compared to those of CA IX in PCa (Fig. 6A,D). This evidence supports the relevance of targeting both extracellular‐facing CA IX and CA XII isoforms as potential therapeutic approaches for PCa treatment. Remarkably, CA IX and CA XII expression was detectable not only in tumor cells (Fig. 6A,D) but also in fibroblasts residing in the tumor milieu, as revealed by a selective antibody staining within the stromal compartment (Fig. 6B,E), indicative of their role in both tumor cells and tumor‐residing fibroblasts. Altogether, these findings provide a clinical relevance to the *in vitro* results, confirming the presence of CA IX and CA XII in both tumor and stromal cells and corroborating their role in tumor progression and worse prognosis in PCa.

## 4 Discussion

The role of ferroptosis in PCa progression has been the focus of several recent preclinical reports and FINs have been proposed as therapeutic approaches for advanced PCa treatment, either individually or in combination with second‐generation antiandrogens [40, 41, 73, 74, 75, 76]. Nevertheless, dissecting the effects of the TME in affecting PCa cell sensitivity to ferroptosis represents an important aspect that needs further elucidation. Indeed, ferroptosis regulation extends beyond the role exerted by cancer cells, being widely affected by several microenvironmental cellular components [32, 77]. In pancreatic ductal adenocarcinoma (PDAC), the transsulfuration pathway activation in CAFs results in enhanced production and secretion of cysteine, which is then uploaded by cancer cells to support ferroptosis resistance [78]. Likewise, ferroportin‐1 and hephaestin upregulation in gastric CAFs are responsible for iron enrichment in the extracellular environment, that is then imported by natural killer cells, promoting ferroptotic death and impairing their antitumor abilities [79]. In accordance with this evidence, we demonstrate in the present study that CAFs support the acquisition of a ferroptosis resistant phenotype in PCa. Specifically, CAF‐secreted metabolites emerged to be involved in the ferroptosis protective mechanism. In agreement, increasing studies underlined that modulation of exogenous nutrient availability strongly alters ferroptosis sensitivity of tumor cells [32, 80]. For instance, exogenous MUFA supplementation suppresses ferroptosis by driving a displacement of PUFAs from being incorporated into membrane phospholipids [81] and reducing exogenous cysteine/cystine through *in vivo* cysteinase administration induces ferroptosis in PDAC [82]. In this scenario, prostate CAFs enhance lactic acid production and secretion in the extracellular milieu [4], thereby impacting PCa cell metabolic reprogramming and aggressiveness [15, 16, 17]. We have previously reported that CAF‐CM has major effects in supporting motility and invasion of PCa cells, while only marginally affecting cell proliferation [15, 16, 83]. Here, we prove that exogenous lactic acid restrains Erastin and RSL3‐induced ferroptosis in PCa cells and inhibits lipid peroxidation. The importance of exogenous lactic acid as a negative ferroptosis regulator aligns with a previous report, demonstrating that lactate uptake/sensing via MCT1 or hydroxycarboxylic receptor 1 results in AMP‐activated protein kinase‐dependent upregulation of steroyl‐CoA desaturase 1, an enzyme involved in the synthesis of the anti‐ferroptotic MUFAs [84]. It is reasonable that certain lipid alterations might also occur in lactic acid‐exposed PCa cells, as this metabolite rewires lipid metabolism [16]. MCT1 targeting exerts significant effects on lactate exchanges *in vivo* [85, 86], representing a potential target to interfere with, and eventually overcome, lactic acid‐induced ferroptosis resistance. Indeed, we underline that MCT1 targeting prevents the exogenous lactic acid‐mediated resistance to ferroptosis in PCa. Interestingly, efficient metastasizing melanoma cells are characterized by high MCT1 levels [86] and melanoma cells transiting through the lymph efficiently metastasize to distant organs by incorporating lymph‐enriched oleic acid, thus preventing ferroptosis [87]. This evidence might support a correlation between MCT1 expression and ferroptosis resistance. In this regard, we reveal a positive correlation between GPX4 and MCT1 expression in a cohort of PCa patients. This finding aligns with our *in vitro* results pointing out that exogenous lactic acid induces the upregulation of GPX4 expression in PCa cells and is in agreement with a recent report underlining higher MCT1 levels in lactate‐rich tissue regions [56]. We also found that GPX4 protein levels increase during PCa progression. Accordingly, protein levels of this ferroptosis marker have been found elevated in adeno‐CRPC patient‐derived xenograft samples from metastatic PCa [40], and in tumor tissues compared to normal ones in several different types of cancer [52, 53, 54]. Beyond their beneficial effects *in vitro* and *in vivo*, utilization of MCT1 inhibitors as primary antitumor treatments in clinical settings (NCT01791595) has raised safety concerns due to MCT1 inhibitor‐induced hyperlactic acidemia [88, 89]. Therefore, identifying novel strategies to impair lactic acid import is necessary to clinically exploit lactic acid‐dependent tumor vulnerabilities. Given that lactic acid secretion is associated with TME acidosis [6, 19], we hypothesized that lactic acid‐induced environmental acidosis could regulate ferroptosis sensitivity in PCa. However, the influence of extracellular acidosis on ferroptosis sensitivity is still controversial. Previous data reported that cells grown at pH 6.5 display increased lipid peroxidation compared to cells maintained at pH 7.4 and supplementation of dietary n‐3 and n‐6 PUFAs induces ferroptosis in acid‐adapted cancer cells by exceeding the PUFA buffering capacity into lipid droplets [33]. Conversely, more recently HIF1α has been reported to drive ferroptosis resistance by mediating the generation of an acidic environment via increased intracellular lactic acid production [90]. In our model, we found that, in accordance with the latter evidence, differently from acidic conditions *per se* created by adding HCl, supporting ferroptosis, the acidic environment generated by exogenous lactic acid induces ferroptosis resistance. Starting from this evidence, we focused our attention on extracellular‐facing CA IX/XII, important regulators of microenvironmental pH [18, 21, 28], as potential novel targets to interfere with CAF‐mediated ferroptosis resistance. Interestingly, CA IX/XII targeting in prostate CAFs leads to a reduction of extracellular acidosis and tumor cells grown in presence of CA IX/XII‐inhibited CAF‐CM are characterized by decreased lactate uptake and enhanced ferroptosis sensitivity, confirming a potential role for extracellular pH in modulating lactate import by cancer cells and consequently in regulating ferroptosis. Given that CA IX/XII inhibitors are administered systemically, we also underline that the direct CA IX/XII targeting in tumor cells disrupts lactic acid exploitation and prevents ferroptosis resistance. According to our findings, previous studies described tumor‐expressed CA IX as a modulator of ferroptosis under hypoxia in breast cancer and malignant mesothelioma [70, 71]. Specifically, in hypoxic breast cancer cells, CA IX targeting, together with depletion of cysteine desulfurase NFS1 or Erastin treatment, increases ferroptosis [91]. Besides, CA IX physically associates with the glutamine transporter solute carrier 1 member 5 (SLC1A5), thereby sustaining an antioxidant response, leading to ferroptosis resistance [92]. Besides directly affecting cancer cells, emerging studies highlighted that the simultaneous increase in acidity and lactate availability within the TME can drive tumor progression by dampening both adaptive and innate antitumor immune responses [93]. In this context, it has been reported that CAF‐secreted lactic acid is uploaded by CD4^+^ T cells, favoring their polarization to Treg and reducing the antitumoral Th1 subpopulation, ultimately promoting cancer cell invasiveness [94]. Further studies underlined that lactate favors the activation and differentiation of Treg in an acidity‐dependent manner [95], while impairing cytotoxicity of CD8^+^ T cells [96]. Moreover, lactic acid, but not the drop in extracellular pH values *per se*, is responsible for the polarization of macrophages toward an anti‐inflammatory phenotype [97] and for the enhancement of the immunosuppressive myeloid‐derived suppressor cells [98]. In this scenario, CA IX/XII targeting, by affecting simultaneously TME acidosis and lactate metabolism, could also be exploited as a therapeutic strategy to switch from the lactic acid‐shaped immunosuppressive environment to an active and efficient immune system. Therefore, although the role of ferroptosis in tumor immunity has not been fully disclosed [99], CA IX/XII inhibition could potentially offer an additional mechanism to synergistically increase tumor sensitivity to ferroptosis by affecting the immune microenvironment. Among the different CA IX/XII inhibitors, sulfonamide‐based compounds, that is, SLC‐0111, are the most successful *in vitro* and *in vivo* [22, 29, 44, 100, 101] and entered clinical trials [46], including a Phase 1b study, in combination with gemcitabine, in CA IX‐positive patients with metastatic PDAC (NCT03450018). These and further studies will hopefully provide key information about the translation of CA IX/XII inhibitor utilization into clinical practice. Interestingly, whereas according to a previous study [72], we found a positive correlation between CA IX expression and PCa grading, for the first time a new role of CA XII has been elucidated in PCa progression, thereby assuming it as a targetable molecule. Finally, in this scenario, we provide preclinical evidence of the efficacy of targeting CA IX/XII in combination with FINs to sensitize PCa cells to ferroptosis.

## 5 Conclusion

In conclusion, this study identifies a ferroptosis susceptibility mechanism in PCa driven by TME factors, including stromal cells, environmental lactic acid, and tumor acidosis. Specifically, lactic acid shuttle within the tumor–stroma crosstalk, cosupported by the activity of the extracellular pH regulators CA IX/XII, is responsible for promoting tumor cell ferroptosis resistance. Notably, CA IX/XII targeting might represent a promising pharmacological approach to disrupt the tumor dependency on lactic acid.

## Conflict of interest

The authors declare no conflict of interest.

## Author contributions

EPa, GC, PC, EG conceived and supervised the study; EPa, GC, EG designed the experiments; EPa, GC, LI, EPr, MI, GG, FV performed the experiments; SB, AN, NL, MB, GN, PS, SS, CTS, AM provided biological samples and reagents; EP, GC, GS analyzed the data; EPa, GC, PC, EG wrote the manuscript; LI, EPr, MI, GS, NL, MB, AM revised the manuscript. All the authors read and approved the final manuscript.

## Peer review

The peer review history for this article is available at https://www.webofscience.com/api/gateway/wos/peer‐review/10.1002/1878‐0261.70083.

## Supporting information


**Fig. S1.** CAF‐secreted lactic acid negatively affects ferroptosis sensitivity in PCa cells.
**Fig. S2.** MCT1 inhibition prevents lactic acid‐induced ferroptosis resistance.
**Fig. S3.** CA IX/XII targeting within the tumor‐stroma crosstalk in PCa avoids lactic acid‐supported ferroptosis resistance.
**Table S1.** Ferroptosis‐related genes up‐ or downregulated in DU145 cells upon lactic acid exposure.

## Data Availability

The data that support the findings of this study are available from the corresponding author (elisa.giannoni@unifi.it) upon reasonable request.
