# Supplementary material for: Targeting carbonic anhydrase IX/XII prevents the anti‐ferroptotic effect of stromal lactic acid in prostate carcinoma
Source: Mol Oncol. 2025 Jun 26;19(9):2515–36. doi: 10.1002/1878-0261.70083 (PMC12420354; doi:10.1002/1878-0261.70083)
Supplement: Supplementary file 1 — Fig. S1. CAF‐secreted lactic acid negatively affects ferroptosis sensitivity in PCa cells. Fig. S2. MCT1 inhibition prevents lactic acid‐induced ferroptosis resistance. Fig. S3. CA IX/XII targeting within the tumor‐stroma crosstalk in PCa avoids lactic acid‐supported ferroptosis resistance. Table S1. Ferroptosis‐related genes up‐ or downregulated in DU145 cells upon lactic acid exposure. [file MOL2-19-2515-s001.zip › Supplementary Table S1_editable.docx]

**Supplementary Table S1.** Ferroptosis-related genes up- or downregulated in DU145 cells upon lactic acid exposure.

| (LA vs CTR) AND (FerrDb) | | | | | | | |
| --- | --- | --- | --- | --- | --- | --- | --- |
| **ENSEMBL** | **SYMBOL** | **GENE_NAME** | **baseMean** | **log2FoldChange** | **stat** | **pvalue** | **padj** |
| ENSG00000104881 | PPP1R13L | protein phosphatase 1 regulatory subunit 13 like | 212.176376 | 0.86864038 | 4.3370152 | 1.44E-05 | 0.00040499 |
| ENSG00000073150 | PANX2 | pannexin 2 | 151.982128 | 1.13395061 | 5.32910564 | 9.87E-08 | 6.78E-06 |
| ENSG00000182054 | IDH2 | isocitrate dehydrogenase (NADP(+)) 2 | 669.998805 | 0.75569051 | 4.52857414 | 5.94E-06 | 0.00019957 |
| ENSG00000185624 | P4HB | prolyl 4-hydroxylase subunit beta | 13311.453 | 0.41778659 | 3.92019082 | 8.85E-05 | 0.00164844 |
| ENSG00000185499 | MUC1 | mucin 1, cell surface associated | 1358.2408 | 1.08817634 | 8.69511528 | 3.46E-18 | 6.01E-15 |
| ENSG00000176108 | CHMP6 | charged multivesicular body protein 6 | 79.6231823 | 1.15602231 | 4.30977671 | 1.63E-05 | 0.00044202 |
| ENSG00000198911 | SREBF2 | sterol regulatory element binding transcription factor 2 | 5417.77176 | 0.38049369 | 2.96995719 | 0.00297841 | 0.02262746 |
| ENSG00000177606 | JUN | Jun proto-oncogene, AP-1 transcription factor subunit | 860.707845 | 0.68949934 | 4.09978752 | 4.14E-05 | 0.00090819 |
| ENSG00000147872 | PLIN2 | perilipin 2 | 1110.56168 | 0.6697757 | 3.86158342 | 0.00011265 | 0.00194725 |
| ENSG00000196139 | AKR1C3 | aldo-keto reductase family 1 member C3 | 129.180222 | 0.76732275 | 3.76117835 | 0.00016911 | 0.00264337 |
| ENSG00000106211 | HSPB1 | heat shock protein family B (small) member 1 | 1053.15805 | 1.43567023 | 5.49889089 | 3.82E-08 | 3.22E-06 |
| ENSG00000167468 | GPX4 | glutathione peroxidase 4 | 3060.07564 | 0.54442515 | 2.63752733 | 0.00835129 | 0.04640348 |
| ENSG00000018510 | AGPS | alkylglycerone phosphate synthase | 1361.63624 | -0.3844939 | -2.976827 | 0.00291248 | 0.02235696 |
| ENSG00000062485 | CS | citrate synthase | 3339.13963 | -0.3371386 | -2.787275 | 0.00531533 | 0.03353493 |
| ENSG00000197601 | FAR1 | fatty acyl-CoA reductase 1 | 1446.09917 | -0.2989983 | -2.6860922 | 0.00722931 | 0.04193183 |
| ENSG00000105993 | DNAJB6 | DnaJ heat shock protein family (Hsp40) member B6 | 2776.05429 | -0.4615106 | -4.4488862 | 8.63E-06 | 0.00026984 |
| ENSG00000111371 | SLC38A1 | solute carrier family 38 member 1 | 11095.6511 | -0.6584511 | -2.9798408 | 0.00288398 | 0.02221402 |
| ENSG00000134324 | LPIN1 | lipin 1 | 3278.94561 | -0.4150241 | -2.6189826 | 0.00881924 | 0.04809866 |
| ENSG00000108733 | PEX12 | peroxisomal biogenesis factor 12 | 97.0021759 | -0.5772927 | -2.6085621 | 0.00909235 | 0.04923989 |
