# Supplementary material for: Targeting carbonic anhydrase IX/XII prevents the anti‐ferroptotic effect of stromal lactic acid in prostate carcinoma
Source: Mol Oncol. 2025 Jun 26;19(9):2515–36. doi: 10.1002/1878-0261.70083 (PMC12420354; doi:10.1002/1878-0261.70083)
Supplement: Supplementary file 1 — Fig. S1. CAF‐secreted lactic acid negatively affects ferroptosis sensitivity in PCa cells. Fig. S2. MCT1 inhibition prevents lactic acid‐induced ferroptosis resistance. Fig. S3. CA IX/XII targeting within the tumor‐stroma crosstalk in PCa avoids lactic acid‐supported ferroptosis resistance. Table S1. Ferroptosis‐related genes up‐ or downregulated in DU145 cells upon lactic acid exposure. [file MOL2-19-2515-s001.zip › Supporting Information.pdf]

Targeting Carbonic Anhydrase IX/XII prevents the anti-ferroptotic effect of stromal lactic acid in prostate carcinoma

Elisa Pardella[1#], Giuseppina Comito[1#], Luigi Ippolito[1], Erica Pranzini[1], Marta Iozzo[1], Giulia Gangarossa[1], Francesca Virgilio[1], Silvia Bua[2], Alessio Nocentini[2], Giada Sandrini[3], Nicla Lorito[1], Marina Bacci[1], Gabriella Nesi[4], Pietro Spatafora[5,6], Sergio Serni[5,6], Claudiu T. Supuran[2], Andrea Morandi[1], Paola Chiarugi[1*], Elisa Giannoni[1*§]

[1] Department of Experimental and Clinical Biomedical Sciences “Mario Serio”, University of Florence, Viale Morgagni 50, 50134, Florence, Italy

[2] Department of NEUROFARBA, Pharmaceutical and Nutraceutical Section, University of Florence, Via Ugo Schiff 6, 50019, Sesto Fiorentino, Florence, Italy

[3] Institute of Oncology Research (IOR), Università della Svizzera Italiana (USI), Via Francesco Chiesa, 6500, Bellinzona, Switzerland

[4] Section of Pathological Anatomy, Department of Health Sciences, University of Florence, Viale Pieraccini 6, 50139, Florence, Italy

[5] Unit of Urological Robotic Surgery and Renal Transplantation, University of Florence, Careggi Hospital, Largo Brambilla 3, 50134, Florence, Italy

[6] Department of Experimental and Clinical Medicine, University of Florence, Largo Brambilla 3, 50134, Florence, Italy

[#] These authors contributed equally as first authors

[*] These authors contributed equally as last authors

[§] Corresponding author: E. Giannoni, elisa.giannoni@unifi.it, Department of Experimental and Clinical Biomedical Sciences “Mario Serio”, University of Florence, Viale Morgagni 50, 50134, Florence, Italy

Supporting Information

Supplementary Table S1. Ferroptosis-related genes up- or downregulated in DU145 cells upon lactic acid exposure.

(LA vs CTR) AND (FerrDb)							
**ENSEMBL**	**SYMBOL**	**GENE_NAME**	**baseMean**	**log2FoldChange**	**stat**	**pvalue**	**padj**
ENSG00000104881	PPP1R13L	protein phosphatase 1 regulatory subunit 13 like	212.176376	0.86864038	4.3370152	1.44E-05	0.00040499
ENSG00000073150	PANX2	pannexin 2	151.982128	1.13395061	5.32910564	9.87E-08	6.78E-06
ENSG00000182054	IDH2	isocitrate dehydrogenase (NADP(+)) 2	669.998805	0.75569051	4.52857414	5.94E-06	0.00019957
ENSG00000185624	P4HB	prolyl 4-hydroxylase subunit beta	13311.453	0.41778659	3.92019082	8.85E-05	0.00164844
ENSG00000185499	MUC1	mucin 1, cell surface associated	1358.2408	1.08817634	8.69511528	3.46E-18	6.01E-15
ENSG00000176108	CHMP6	charged multivesicular body protein 6	79.6231823	1.15602231	4.30977671	1.63E-05	0.00044202
ENSG00000198911	SREBF2	sterol regulatory element binding transcription factor 2	5417.77176	0.38049369	2.96995719	0.00297841	0.02262746
ENSG00000177606	JUN	Jun proto-oncogene, AP-1 transcription factor subunit	860.707845	0.68949934	4.09978752	4.14E-05	0.00090819
ENSG00000147872	PLIN2	perilipin 2	1110.56168	0.6697757	3.86158342	0.00011265	0.00194725
ENSG00000196139	AKR1C3	aldo-keto reductase family 1 member C3	129.180222	0.76732275	3.76117835	0.00016911	0.00264337
ENSG00000106211	HSPB1	heat shock protein family B (small) member 1	1053.15805	1.43567023	5.49889089	3.82E-08	3.22E-06
ENSG00000167468	GPX4	glutathione peroxidase 4	3060.07564	0.54442515	2.63752733	0.00835129	0.04640348
ENSG00000018510	AGPS	alkylglycerone phosphate synthase	1361.63624	-0.3844939	-2.976827	0.00291248	0.02235696
ENSG00000062485	CS	citrate synthase	3339.13963	-0.3371386	-2.787275	0.00531533	0.03353493
ENSG00000197601	FAR1	fatty acyl-CoA reductase 1	1446.09917	-0.2989983	-2.6860922	0.00722931	0.04193183
ENSG00000105993	DNAJB6	DnaJ heat shock protein family (Hsp40) member B6	2776.05429	-0.4615106	-4.4488862	8.63E-06	0.00026984
ENSG00000111371	SLC38A1	solute carrier family 38 member 1	11095.6511	-0.6584511	-2.9798408	0.00288398	0.02221402
ENSG00000134324	LPIN1	lipin 1	3278.94561	-0.4150241	-2.6189826	0.00881924	0.04809866
ENSG00000108733	PEX12	peroxisomal biogenesis factor 12	97.0021759	-0.5772927	-2.6085621	0.00909235	0.04923989

Supplementary Figure S1

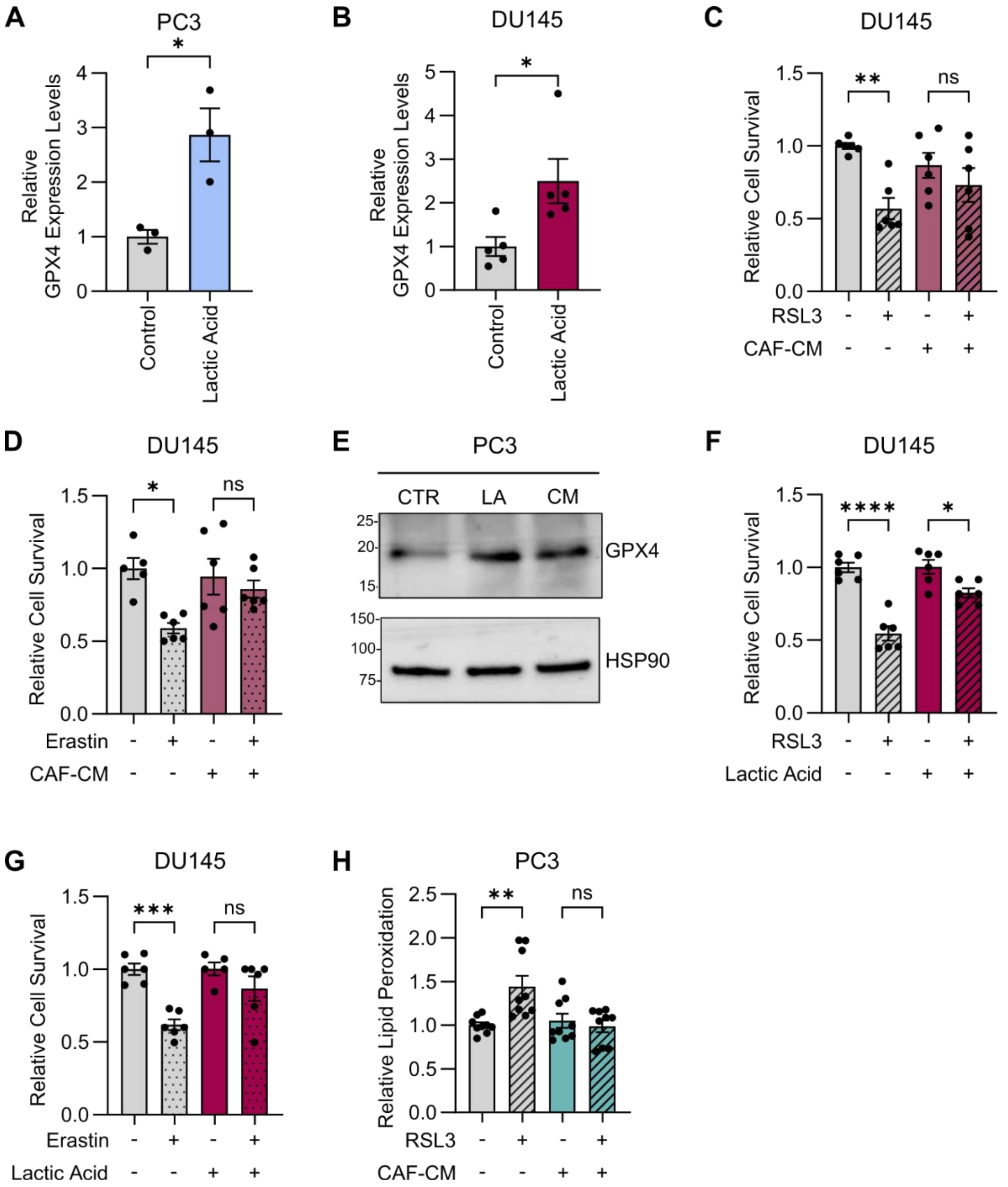

Supplementary Figure S1. CAF-secreted lactic acid negatively affects ferroptosis sensitivity in PCa cells. A,B) Western blot signal quantification through densitometric analysis representing GPX4 protein expression in PC3 (**A**) and DU145 (**B**) cells treated for 48 hours with serum-free medium (Control) or 20 mM lactic acid. HSP90 was utilized as loading control. Relative protein expression is reported using serum-free medium-treated cells as comparator (A n = 3; B n = 5 biologically-independent replicates). **C,D)** Cell viability was assessed in DU145 cells incubated with serum-free medium or CAF-CM for 24 hours and then treated for additional 24 hours with 1 μM RSL3 (**C**) or 10 μM Erastin (**D**) (n = 3 biologically-independent replicates, each performed in duplicate technical replicates). **E)** GPX4 protein levels were evaluated by western blot analysis on total protein lysates from PC3 cells treated with serum-free medium (CTR) or 20 mM lactic acid (LA) or CAF-CM

(CM) for 48 hours. HSP90 was used as loading control. Representative images of at least three independent experiments are reported. **F,G)** Cell viability was evaluated in DU145 cells incubated with serum-free medium or 20 mM lactic acid for 24 hours and then treated with 1 μM RSL3 (**F**) or 10 μM Erastin (**G**) for additional 24 hours (n = 3 biologically-independent replicates, each performed in duplicate technical replicates). **H)** PC3 cells were treated with serum-free medium or CAF-CM for 24 hours before incubation with RSL3. Lipid peroxidation levels were measured by staining cells with 5 μM C11-BODIPY$^{(581/591)}$ for 30 minutes at 37°C and then performing fluorimetric analysis (n = 3 biologically-independent replicates, each performed in triplicate technical replicates). Relative cell survival (**C,D,F,G**) or relative lipid peroxidation (**H**) is reported using serum-free medium-treated cells as comparator. In **A-D, F-H,** data are reported as mean ± SEM of n biological independent experiments, each performed with the indicated technical replicates. Statistical analyses were performed using two-tailed unpaired Student’s t-test (**A,B**) or one-way ANOVA followed by Tukey’s multiple comparisons test (**C,D, F-H**). ns, not significant; * $p < 0.05$; ** $p < 0.01$; *** $p < 0.001$; **** $p < 0.0001$.

Supplementary Figure S2

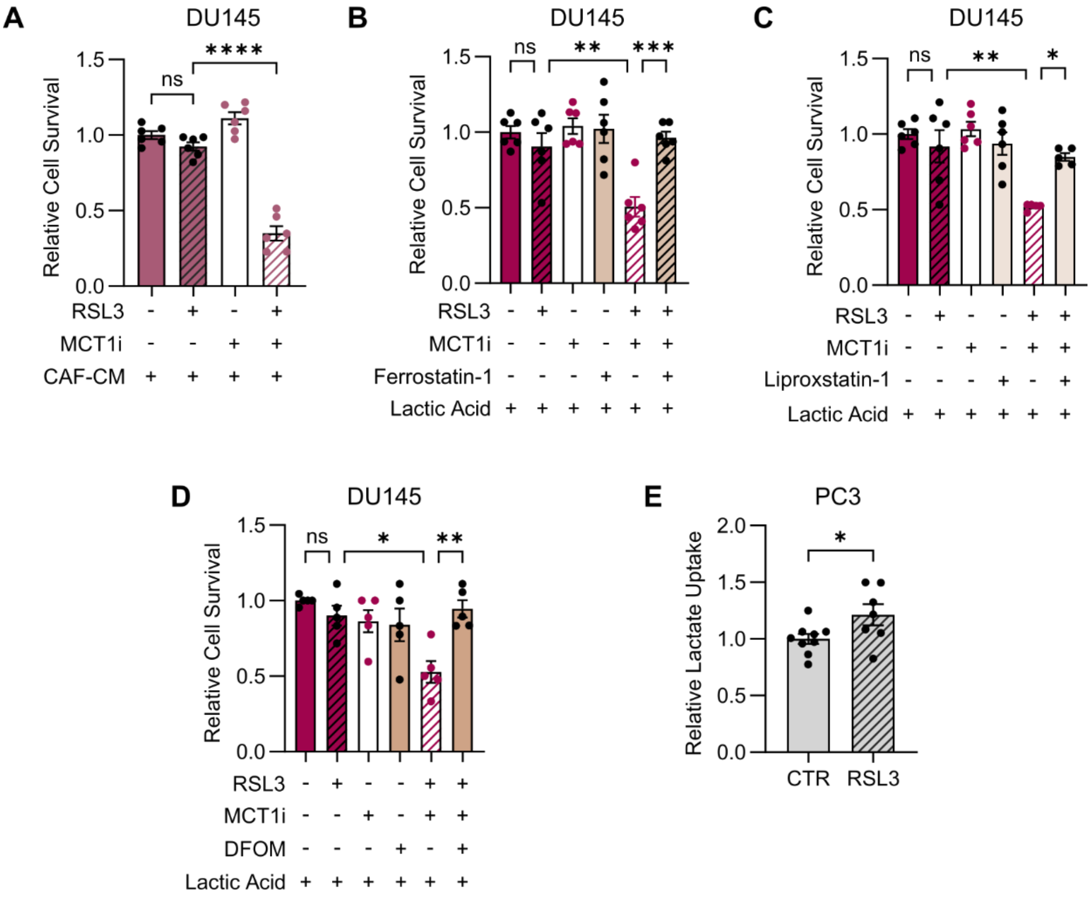

Supplementary Figure S2. MCT1 inhibition prevents lactic acid-induced ferroptosis resistance. A) Cell viability was measured in DU145 cells exposed to CAF-CM and 40 μM MCT1 inhibitor (MCT1i) AR-C155858 for 24 hours, and then to 1 μM RSL3 for additional 24 hours. Relative cell survival is reported using CAF-CM-treated cells as comparator (n = 3 biologically-independent replicates, each performed in duplicate technical replicates). **B-D)** DU145 cells were exposed for 24 hours to 20 mM lactic acid and 40 μM MCT1i AR-C155858. Then, cells were pre-treated for 2 hours with 1 μM Ferrostatin-1 (**B**) or 1 μM Liproxstatin-1 (**C**) or 10 μM DFOM (**D**) before incubation with 1 μM RSL3 for 24 hours. Then, cell viability was assessed. Relative cell survival is reported using lactic acid-treated cells as comparator (n = 3 biologically-independent replicates, each performed in single or duplicate technical replicates). **E)** PC3 cells were treated for 24 hours with serum-free medium supplemented or not with 1 μM RSL3. Then, the incorporation of exogenous ^{14}C-lactate was measured and normalized on protein content (n = 3 biologically-independent replicates, each performed with 2-4 technical replicates). Relative lactate uptake is reported using serum-free medium-treated cells as comparator. In **A-E**, data are reported as mean ± SEM of n biological independent experiments, each performed with the indicated technical replicates. Statistical analyses were performed using one-way ANOVA followed by Dunnett's multiple comparisons test

(**A**), Tukey's multiple comparisons test (**B-D**), or two-tailed unpaired Student's t-test (**E**). ns, not significant; * $p < 0.05$; ** $p < 0.01$; *** $p < 0.001$; **** $p < 0.0001$.

Supplementary Figure S3

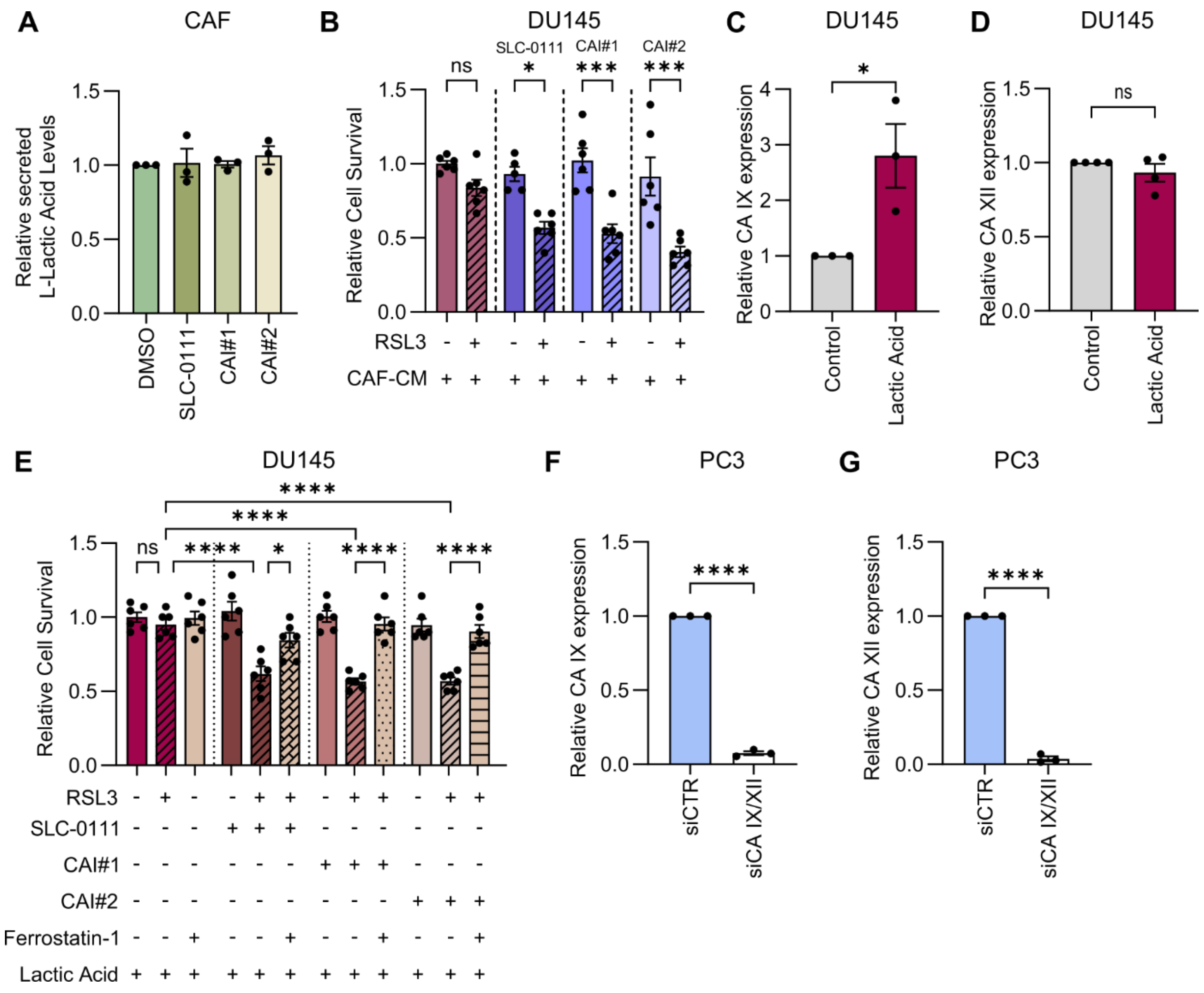

Supplementary Figure S3. CA IX/XII targeting within the tumor-stroma crosstalk in PCa avoids lactic acid-supported ferroptosis resistance. A) L-Lactic Acid levels measured in extracellular culture media isolated from CAFs, that were previously treated for 48 hours with serum-free medium in the presence or not of 2 µM CA IX/XII inhibitors (SLC-0111, CAI#1, CAI#2) (n = 3 biologically-independent replicates). Untreated CAFs were used as comparator. **B)** Cell viability was evaluated in DU145 cells treated for 24 hours with CM isolated from CAFs, previously incubated with 2 µM CA IX/XII inhibitors (SLC-0111, CAI#1, CAI#2), and for additional 24 hours with 1 µM RSL3. Relative cell survival is reported using CAF-CM-treated cells as comparator (n = 3 biologically-independent replicates, each performed in duplicate technical replicates). **C, D**) mRNA levels of CA IX (**C**) and CA XII (**D**) evaluated by qRT-PCR in DU145 cells exposed for 48 hours to serum-free medium (Control) or 20 mM lactic acid (C, n = 3; D, n = 4 biologically-independent replicates). Relative expression is reported using control cells as comparator. **E)** DU145 cells were exposed for 24 hours to 20 mM lactic acid and 2 µM CA IX/XII inhibitors (SLC-0111, CAI#1, CAI#2). Then, cells were pre-treated for 2 hours with 1 µM Ferrostatin-1 before incubation with 1 µM RSL3 for 24 hours. Then, cell viability was evaluated. Relative cell survival is reported using lactic acid-treated cells as comparator (n = 3

biologically-independent replicates, each performed in duplicate technical replicates). **F,G)** mRNA levels of CA IX (**F**) and CA XII (**G**) evaluated by quantitative RT-PCR in PC3 cells transiently silenced using non-targeting control (siCTR) or CA IX and CA XII selective combo siRNA (siCA IX/XII) and then exposed for 48 hours to 20 mM lactic acid (n = 3 biologically-independent replicates). Relative expression is reported using siCTR cells as comparator. In **A-G**, data are reported as mean ± SEM of n biological independent experiments, each performed with the indicated technical replicates. Statistical analyses were performed using one-way ANOVA followed by Dunnett's multiple comparison test (**A**) or Tukey's multiple comparisons test (**B, E**) or two-tailed unpaired Student's t-test (**C,D,F,G**). ns, not significant; * $p < 0.05$; *** $p < 0.001$; **** $p < 0.0001$.
